# Genome Sequencing of a Gray Wolf from Peninsular India Provides New Insights into the Evolution and Hybridization of Gray Wolves

**DOI:** 10.1093/gbe/evac012

**Published:** 2022-02-08

**Authors:** Ming-Shan Wang, Mukesh Thakur, Yadvendradev Jhala, Sheng Wang, Yellapu Srinivas, Shan-Shan Dai, Zheng-Xi Liu, Hong-Man Chen, Richard E Green, Klaus-Peter Koepfli, Beth Shapiro

**Affiliations:** 1 Howard Hughes Medical Institute, University of California Santa Cruz, USA; 2 Department of Ecology and Evolutionary Biology, University of California Santa Cruz, USA; 3 Zoological Survey of India, New Alipore, Kolkata, West Bengal, India; 4 State Key Laboratory of Genetic Resources and Evolution, Kunming Institute of Zoology, Chinese Academy of Sciences, Kunming, China; 5 Wildlife Institute of India, Chandrabani, Dehradun, Uttarakhand, India; 6 College of Animal Science, Jilin University, Changchun, China; 7 College of Animal Science and Technology, Yunnan Agricultural University, Kunming, China; 8 Department of Biomolecular Engineering, University of California Santa Cruz, USA; 9 Smithsonian-Mason School of Conservation, George Mason University, USA; 10 Center for Species Survival, Smithsonian Conservation Biology Institute, National Zoological Park, Washington, District of Columbia, USA; 11 Computer Technologies Laboratory, ITMO University, St. Petersburg, Russia

**Keywords:** gray wolf, *Canis lupus pallipes*, hybridization, gene flow, evolutionary history, Pleistocene refugium

## Abstract

The gray wolf (*Canis lupus*) is among the few large carnivores that survived the Late Pleistocene megafaunal extinctions. Thanks to their complex history of admixture and extensive geographic range, the number of gray wolf subspecies and their phylogenetic relationships remain poorly understood. Here, we perform whole-genome sequencing of a gray wolf collected from peninsular India that was phenotypically distinct from gray wolves outside India. Genomic analyses reveal that the Indian gray wolf is an evolutionarily distinct lineage that diverged from other extant gray wolf lineages ∼110 thousand years ago. Demographic analyses suggest that the Indian wolf population declined continuously decline since separating from other gray wolves and, today, has exceptionally low genetic diversity. We also find evidence for pervasive and mosaic gene flow between the Indian wolf and African canids including African wolf, Ethiopian wolf, and African wild dog despite their current geographical separation. Our results support the hypothesis that the Indian subcontinent was a Pleistocene refugium and center of diversification and further highlight the complex history of gene flow that characterized the evolution of gray wolves.

Significance The gray wolf (*Canis lupus*) is one of the few megafaunal carnivores that survived the Late Pleistocene megafaunal extinctions. Despite extensive research on living and extinct gray wolves, the evolutionary history of this lineage remains unclear. Here, we sequence and analyze a draft genome of a gray wolf collected from peninsular India. We find that the Indian wolf lineage, which is both highly threatened and phenotypically distinct from other gray wolves, is the most deeply diverging lineage of extant gray wolves and, despite their physical isolation from other wolf lineages, has a long history of gene flow with other canid lineages.

## Introduction

The gray wolf (*Canis lupus*) first appears in the fossil records of Eurasia and North America some 500,000 years ago (Nowak 1979) and later diversified into more than 37 named subspecies (Wilson and Reeder 2005). Numerous morphological and genomic analyses of gray wolves have presented a complex and sometimes contradictory view of their evolutionary history (Leonard et al. 2007; Sinding et al. 2018; Smeds et al. 2019; Loog et al. 2020). For example, analyses of mitochondrial DNA have revealed a lack of strong haplotype structure among populations across the Northern hemisphere (Thalmann et al. 2013; Loog et al. 2020), whereas nuclear genomic analyses have identified distinct lineages in Eurasia and North America (Fan et al. 2016; Gopalakrishnan et al. 2018). These studies have also revealed widespread admixture among domestic dogs, gray wolves, and other species in the genera *Canis* and *Cuon* (Freedman et al. 2014; Fan et al. 2016; Gopalakrishnan et al. 2018; Pilot et al. 2019). This evolutionary history of dynamic long-distance dispersal, population replacements, and cross-species gene flow has complicated efforts to understand both how gray wolf populations are related to each other and the location, origin, and timing of dog domestication (Koepfli et al. 2015; Perri et al. 2021).

Among the least studied populations of gray wolves are those that inhabit the Indian subcontinent. Early taxonomists described two species endemic to this region: the Himalayan wolf, *Canis laniger* (Hodgson 1847), found in the highland regions of the Tibetan Plateau and eastern Kashmir, and the Indian wolf *Canis pallipes* (Sykes 1831), distributed within the arid/semi-arid lowland plains of peninsular India. Since these first descriptions, Himalayan and Indian wolves have been reclassified as subspecies within the gray wolf complex, *Canis lupus chanco* and *C. l. pallipes*, respectively (Allen 1938). The current range of *C. l. pallipes* extends from the eastern Mediterranean region of western Asia eastward to peninsular India, where several isolated populations are reported (Nowak 1995; Jhala 2003).

**Fig. 1.**
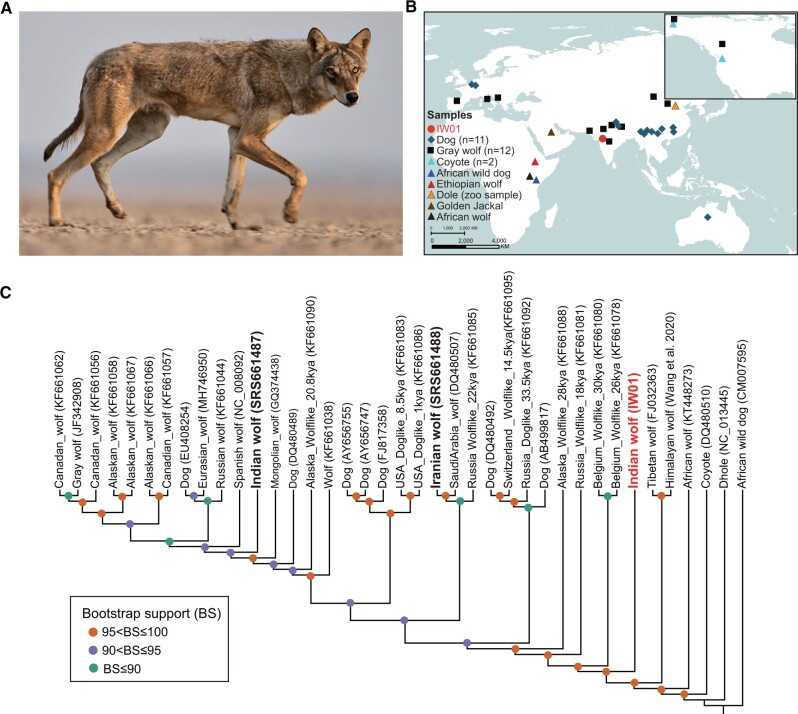
Sampling location and mitochondrial phylogeny. (*A*) A photograph of an Indian wolf from peninsular India (provided by Y. Shah). (*B*) Map showing the distribution of samples used in this study. The red dot depicts the location where the Indian wolf IW01 (supplementary fig. S1, Supplementary Material online) was sampled. (*C*) Maximum-likelihood tree estimated from mitochondrial genomes (15,462 bp). The Indian wolf (IW01) is a sister clade to domestic dogs and other gray wolves but inside the lineage of Tibetan wolf+Himalayan wolf. IDs in brackets are the GenBank accession numbers.

Genetic studies using mitochondrial and nuclear markers have shown that the Himalayan wolf is distinct from other gray wolf populations (Aggarwal et al. 2007; Ersmark et al. 2016; Werhahn et al. 2017, 2018). Similarly, Indian wolves are morphologically, behaviorally, and genetically distinct from other wolf subspecies (Aggarwal et al. 2003; Sharma et al. 2004; Aggarwal et al. 2007). Compared with other wolves, Indian wolves are smaller in size (18–22 kg) with less and relatively shorter hair that is light brown in color with black hair tips (fig. 1*A*). Indian wolves are also among the most threatened canid subspecies in the world, with an estimated population size of ∼2,000–3,000 individuals (Jhala 2003).

More recently, relationships among gray wolves have been analyzed using whole-genome sequences. In one study examining admixture among gray wolves and domestic dogs (Fan et al. 2016), a wolf presumed to originate from India but lacking precise locality information (NCBI accession: SRS661487) clustered with wolves from Iran and Israel, which together were grouped within a larger cluster of gray wolves from Eurasia. This result was, however, at odds with earlier phylogenetic analyses based on mitochondrial sequences that suggested that Himalayan wolves and wolves from peninsular India are the earliest branchings and most divergent lineages among all gray wolf populations, with Indian wolves diverging from other lineages ∼270–400 thousand years ago (ka) (Aggarwal et al. 2003; Sharma et al. 2004; Aggarwal et al. 2007). To resolve this inconsistency, additional analyses using wolf samples of unambiguous provenance are necessary, in particular as the complex history of admixture among canids can lead to discordance among individual gene trees (mitochondrial and nuclear) and the population/species tree (Degnan and Rosenberg 2009; Toews and Brelsford 2012).

Here, we address this by generating and analyzing a high-coverage nuclear and mitochondrial genome from a male Indian wolf captured for a radio-telemetry study in Velavadar Blackbuck National Park, Gujarat State, in western India (Jhala 2003), which we call IW01. IW01 had the morphological traits (supplementary fig. S1, Supplementary Material online) and mitochondrial sequence of a typical Indian peninsular wolf (Sharma et al. 2004). We analyze IW01 in conjunction with previously published genomic data from gray wolves sampled from Eurasia and North America (supplementary table S1, Supplementary Material online), including SRS661487, the wolf mentioned above whose precise origins remain ambiguous. We find strong evidence that IW01, along with Himalayan/Tibetan wolves, comprise lineages that are basal to all other gray wolves in both mitochondrial and nuclear phylogenies. Reconstruction of demographic histories also reveals that IW01 has a distinct effective population size trajectory compared with other wolves. Finally, we uncover evidence of historical admixture between IW01 and several canid lineages from Africa despite their current geographical separation, as well as gene flow between the domestic dog + gray wolf clade and these African canids. Our analyses indicate, however, that despite this history of admixture, the Indian wolf lineage has been evolving in isolation from other gray wolf lineages for around 110 thousands years.

## Results and Discussion

### Genome Sequencing and Mitochondrial Phylogeny

We extracted genomic DNA from IW01 using a whole blood sample collected in 1995. We prepared four pair-end sequencing libraries from which we sequenced 93.5 G nucleotide bases. We mapped sequencing reads to the domestic dog CanFam3.1 reference genome assembly, which yielded a 30.7-fold coverage genome for IW01. In addition, we de novo assembled the mitochondrial genome from IW01 to 2,557-fold coverage. From this whole mitochondrial genome, we extracted the cytochrome *b* and 16S rRNA gene sequences, which we used to estimate a phylogeny including IW01 and previously published mitochondrial data from Indian and other gray wolves for which full mitochondrial genomes were unavailable. Maximum-likelihood trees based on these two genes place IW01 in a previously reported clade containing other wolves from peninsular India that, along with Himalayan/Tibetan wolves, is basal to Holarctic gray wolves and domestic dogs (supplementary fig. S2, Supplementary Material online).

Using published raw read data, we also de novo assembled mitochondrial genomes of wolves putatively originating from India (SRS661487) and Iran (SRS661488), both of which lack precise locality information (Fan et al. 2016). We aligned these to a data set of 36 previously published mitochondrial genomes representing different Eurasian and North American gray wolf populations, including one Tibetan wolf and one Himalayan wolf, domestic dogs, and other species belonging to the genera *Canis*, *Cuon*, and *Lycaon*. As with the single gene analyses, IW01 was basal to all Holarctic gray wolves but inside the clade containing the Himalayan and Tibetan wolves, and distant from the SRS661487 (India) and SRS661488 (Iran), which cluster within the clade comprising Holarctic wolves and domestic dogs (fig. 1*C*).

### Phylogenetic Relationship between the Indian Wolf IW01 and Other Gray Wolves

**Fig. 2.**
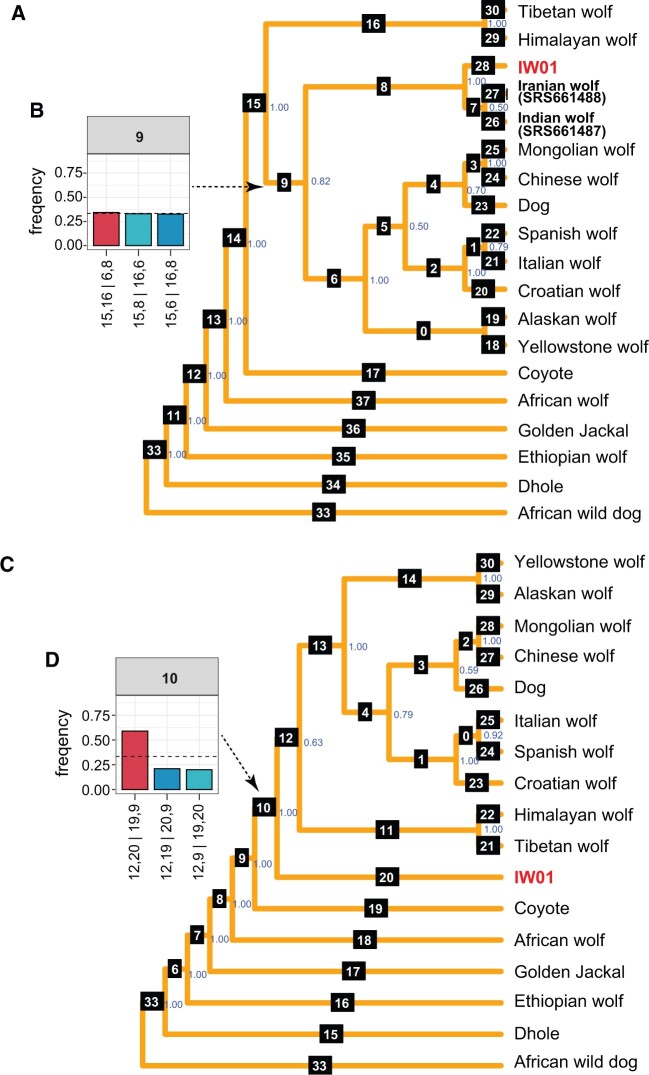
Phylogenetic analysis of Indian wolf IW01 and other canids based on nuclear genomes. (*A*) Consensus phylogenetic tree obtained using ASTRAL-III, estimated from 5,000 20-kb regions sampled across the nuclear genomes of the domestic dog, representative gray wolves, and other canid species. The blue-colored values at each node show the mean posterior probability of that node. White numbers with black squares denote branch numbers. As domestic dogs are a monophyletic group within the clade containing gray wolves from Eurasia (Fan et al. 2016; Wang et al. 2016), we only chose one high-coverage domestic dog genome for this analysis. (*B*) Quartet frequencies of three possible topologies for branch 9 in (*A*). The format “15,16|6,8” indicates the quartet topology with branches 15 and 16 together on one side and branches 6 and 8 on the other side. Quartet topology frequencies for 16 branches in the underlying unrooted phylogeny are shown in supplementary figure S3*A*, Supplementary Material online. The red bar indicates the frequency of the topology shown in (*A*) and the other blue-colored bars represent frequencies of the two alternative topologies. The dotted line represents the one-third frequency cut-off of the true topology for each quartet (Allman et al. 2011). (*C*) Phylogenetic tree estimated from the nuclear genome using ASTRAL-III but excluding the previously reported gray wolf genomes of SRS661487 (India) and SRS661488 (Iran). (*D*) The quartet frequencies of three possible topologies for branch 10 in (*C*). Quartet topology frequencies for 14 branches in the underlying unrooted phylogeny are shown in supplementary figure S3*B*, Supplementary Material online.

We combined gene trees estimated from 5,000 randomly selected 20-kb regions across the nuclear genomes of IW01 and 18 other canids and reconstructed a species tree using ASTRAL-III (Zhang et al. 2018). As observed previously (Koepfli et al. 2015; Gopalakrishnan et al. 2018), the African wolf and golden jackal are basal to the coyote and gray wolf clades (fig. 2*A* and *B*), and the Ethiopian wolf is an outgroup to the golden jackal. Domestic dogs and East Asian gray wolves formed a clade sister to European gray wolves, but with low support (fig. 2*A* and supplementary fig. S3*A*, Supplementary Material online). Quartet frequencies of gene trees comprising domestic dog, East Asian wolf, and European wolf were similar (supplementary fig. S3*A*, Supplementary Material online). When IW01, SRS661487 (India), and SRS661488 (Iran) are included in the ASTRAL tree, these three lineages form a well-supported clade basal to North American and Eurasian wolves following the split of Himalayan and Tibetan wolves, the latter of which comprises the earliest diverging lineage in the gray wolf/domestic dog clade (fig. 2*A*). This result is inconsistent with the phylogenetic tree presented in Fan et al. (2016), based on a supermatrix analysis of genome-wide SNP data that do not account for gene tree discordance. In Fan et al. (2016), SRS661487 and SRS661488 fall in the clade with European wolves, as they do in our mitochondrial phylogeny (fig. 1*C*). When we estimated the ASTRAL tree excluding IW01, SRS661487, and SRS661488 cluster with European wolves (supplementary fig. S4, Supplementary Material online) as in Fan et al. (2016). When the ASTRAL tree includes IW01 but excludes SRS661487 and SRS661488, IW01 falls basal to all gray wolves and the domestic dog, including the Himalayan and Tibetan wolf clade, with strong support (fig. 2*C* and *D*, panel 10). However, the placement of the Himalayan/Tibetan wolf clade has low support (supplementary fig. S3*B*, Supplementary Material online), suggesting that the phylogenetic relationship among IW01, Himalayan/Tibetan wolf, and the domestic dog + gray wolf clade is not well resolved, possibly due to incomplete sorting and/or gene flow among these lineages.

To further explore the placement of IW01, we aligned the high-coverage nuclear genomes from IW01, a Tibetan wolf, a Chinese wolf, and a dhole and divided the alignment into 250-kb, 500-kb, and 1-Mb nonoverlapping segments, and then estimated maximum-likelihood phylogenetic trees for each segment. The most commonly observed topology, which accounted for 48–57% of windows, placed IW01 as basal to the Tibetan wolf and the Chinese wolf (supplementary fig. S5, Supplementary Material online).

Given that the most commonly observed topology placed IW01 as basal to Tibetan wolves, which previously estimated contained as much as 39% ancestry from a deeply divergent “ghost” lineage (Wang et al. 2020), it is possible that all or some component of the ancestry of IW01 is also from this “ghost” lineage. To test this, we constructed a neighbor-joining tree using only genomic segments characterized as of “ghost” origin in Himalayan and Tibetan wolves (Wang et al. 2020). Similar to the mitochondrial tree (fig. 1*C*), IW01 and Himalayan/Tibetan wolves formed two distinct clades in this analysis, with the latter clade basal to other gray wolves, including IW01, with high bootstrap support (supplementary fig. S6, Supplementary Material online). These results suggest that IW01 is not the possible source of the “ghost” lineage ancestry. Instead, the “ghost” lineage is likely basal to IW01.

**Fig. 3.**
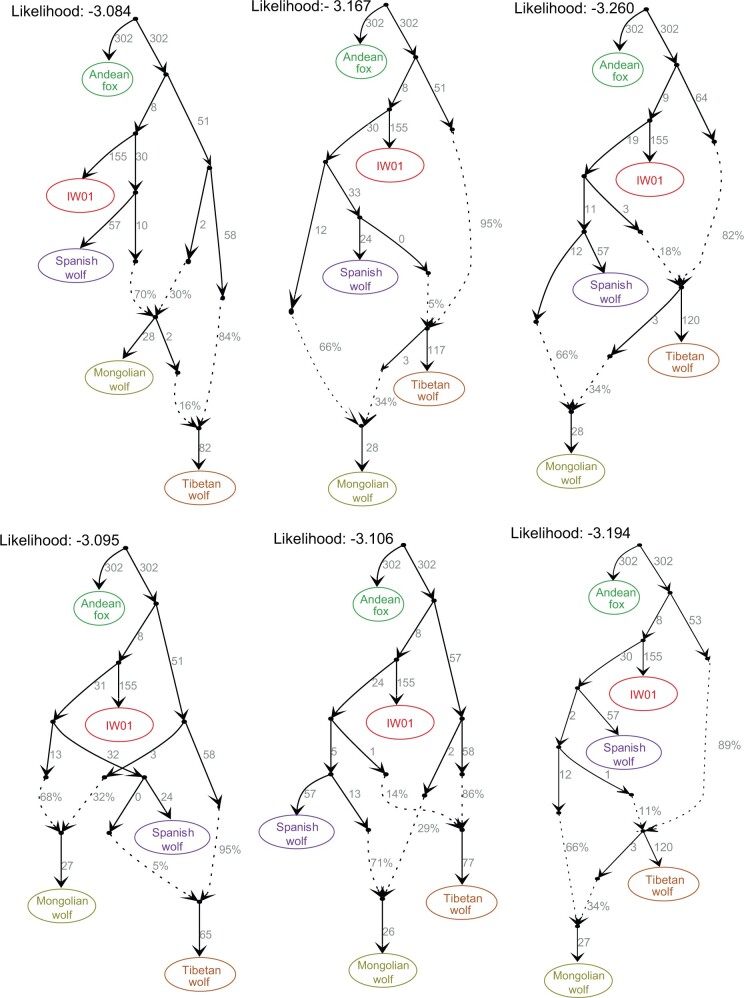
Fitted admixture graphs (no f4 outliers) showing the genetic makeup for IW01, European wolves (represented here by the Spanish wolf), and two highland wolves (represented by the Mongolian wolf and Tibetan wolf). Dashed lines indicate inferred admixture events and the admixture proportions are reported next to the dashed lines. The likelihood is shown at the top of each graph. The first graph has the highest likelihood of support.

Finally, we modeled the genetic makeup and phylogenetic assignments of IW01 using admixture graphs. Because this analysis is based on genotype calls, we prioritized genomes with sequence coverage over 10-fold. In agreement with the above analyses, our data fit the graph models (no f4 outliers) in which IW01 is assigned to a lineage basal to Eurasian gray wolves and shows no signals of admixture with other gray wolf populations (fig. 3). Our results also indicate Tibetan wolves have admixed ancestry that is perhaps derived from ancient hybridization between a lineage basal to IW01 and Eurasian gray wolves. Interestingly, this analysis suggests that the Mongolian wolf is also admixed (fig. 3), with the majority of its ancestry coming from European wolves, and the remainder from a lineage connecting them to Himalayan/Tibetan wolves. Previous studies have suggested that the range of Himalayan/Tibetan wolves was probably expanded across much of Mongolia and Northwest China (Sharma et al. 2004), although these wolves maintain different distributions and represent distinct genetic lineages today (Werhahn et al. 2017).

### Gene Flow between Indian Wolf IW01 and Other Canids

**Fig. 4.**
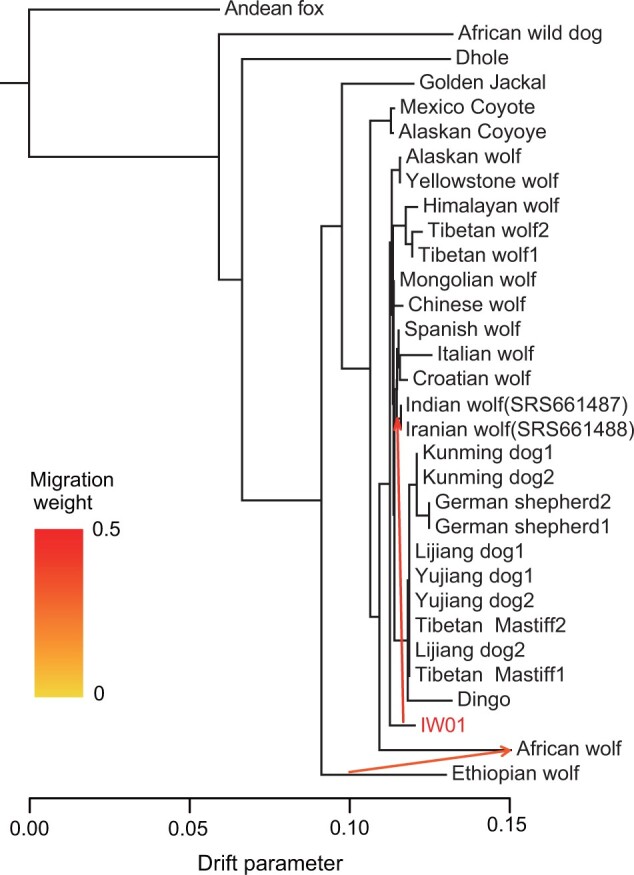
TreeMix tree graph allowing two migration edges. This configuration reveals IW01 is basal to other gray wolves and domestic dogs. Two admixture events are shown, one between the African wolf and Ethiopian wolf, and the other between IW01 and SRS661487 (India)+SRS661488 (Iran). Tree graphs and Treemix residuals inferred by allowing zero to five migration edges are shown in supplementary figures S7 and S8, Supplementary Material online. The graph with two migrations has the lowest residual distance.

The uncertainty of the phylogenetic placement of gray wolves SRS661487 (India) and SRS661488 (Iran), as well as previous reports of admixture among canid lineages (Koepfli et al. 2015; Skoglund et al. 2015; Gopalakrishnan et al. 2018), suggest that one or more of the sampled Middle Eastern and Indian wolf lineages may have admixed ancestry. We explored genetic affinity and admixture between IW01 and other gray wolves using TreeMix (Pickrell and Pritchard 2012) and *D*-statistics (Green et al. 2010) by analyzing 32 nuclear genomes (supplementary table S1, Supplementary Material online). Our results support IW01 as a diverged wolf lineage basal to other Eurasian gray wolves and that SRS661487 is closely related to Iranian and European gray wolves (fig. 4 and supplementary figs. S4–S9, Supplementary Material online).

**Fig. 5.**
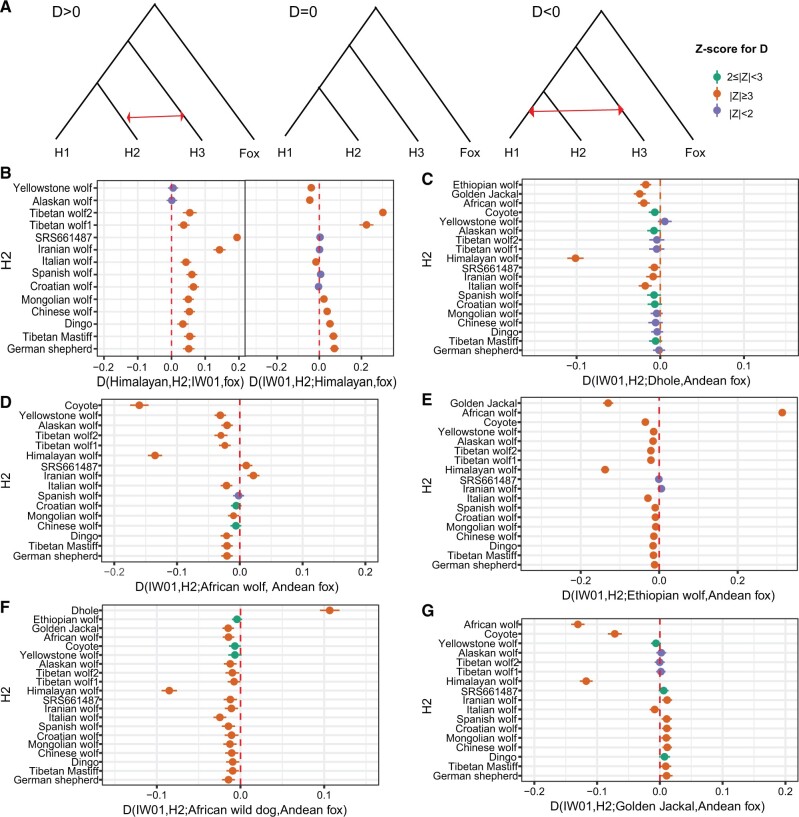
D-statistics testing the amount of allele sharing between IW01 and other canid species. (*A*) Schematic plot showing the topology used for calculating *D*-statistics. The calculation with |*Z*|≥3 was considered statistically significant. (*B*) *D*-statistics find no clear evidence of admixture between IW01 and Himalayan wolf. Most *D* values are positive, suggesting that IW01 shares more derived alleles with other gray wolves (H2) than with the Himalayan wolf. We note that D(IW01, H2; Himalayan wolf, Andean fox) calculated when H2 includes North American wolves showed significant negative values. However, this should not be taken to signify admixture between IW01 and the Himalayan wolf. Rather, this is likely due to North American wolves sharing ancestry from more divergent species like the coyote (vonHoldt et al. 2016; Sinding et al. 2018). (*C*) *D*-statistics plot showing the amount of allele sharing between dhole and IW01 and other gray wolves. (*D*) *D*-statistics plot showing allele sharing between the African wolf and IW01. (*E*) *D*-statistics plot depicting allele sharing between the Ethiopian wolf and IW01. This test also finds evidence of admixture between the African wolf and the Ethiopian wolf. (*F*) *D*-statistics plot showing allele sharing between the African wild dog and IW01. (*G*) *D*-statistics plot showing the amount of allele sharing between the golden jackal and the ancestral clade of gray wolves. *D*-statistics were significantly positive when the domestic dog, East Asian wolves, and European wolves were in position H2, but became insignificant when North American wolves and Tibetan wolves were in the H2 position. These results suggest past gene flow between the Eurasian golden jackal and the ancestor of domestic dog and Eurasian gray wolves, supporting previous studies (Gopalakrishnan et al. 2018; Chavez et al. 2019). This also suggests that the Eurasian golden jackal has no or less gene flow with IW01 compared with domestic dogs and Eurasian gray wolves, despite the overlapping distributions of the former two species.

We also found evidence of gene flow between IW01 and the two wolves of suspect origin (SRS661487 [India] and SRS661488 [Iran]) (figs. 4 and 5*A* and *B*; supplementary figs. S7–S9, Supplementary Material online), as well as between IW01 and three more recently reported Iranian wolves (supplementary fig. S10, Supplementary Material online) (Amiri Ghanatsaman et al. 2020). Admixture among these lineages is expected, given the lack of reproductive barriers and any major geographic barriers separating these populations. We did not find evidence, however, of gene flow between IW01 and the Himalayan wolf. This is surprising, given the proximity of their ranges but consistent with previous findings based on mitochondrial sequences (Sharma et al. 2004) and our phylogenetic results. It is possible that differences in local adaptation between highland wolves of the trans-Himalayan and Tibetan plateau (Werhahn et al. 2018; Wang et al. 2020) versus lowland wolves of the semi-arid habitats in peninsular India, along with the small population sizes and fragmented habitat of Indian wolves may lessen chances for admixture between these lineages (Owen et al. 2002; Blinkhorn and Petraglia 2017). However, given that our analyses are currently limited to a single Indian wolf sample of known origin, additional genomes from wolves sampled across peninsular India and the Himalayan region will be required to reveal the extent of gene flow among these lineages.

Using *D*-statistics, we did not find any evidence of admixture between IW01 and the Asiatic dhole when the domestic dog, East Asian wolf, Croatian wolf, Spanish wolf, or North American wolf are in position H2 (fig. 5*C*). However, we detected significant gene flow between IW01 and Kenyan African wolf (fig. 5*D*), Ethiopian wolf (fig. 5*E*), and African wild dog (fig. 5*F*). This is consistent with the recent radiation of including *Lycaon*, *Cuon*, and *Canis*, which has been estimated at ∼1.72 Ma in models that include the possibility of gene flow among lineages (Chavez et al. 2019). Such gene flow may have been mediated through an unknown, earlier diverging donor species (Gopalakrishnan et al. 2018). We also found evidence of gene flow between IW01 and each of three recently reported northwestern African wolves (from Senegal, Morocco, and Algeria) (Liu et al. 2018), although the proportion of shared ancestry varied among individuals sampled (supplementary tables S2 and S3, Supplementary Material online). Moreover, past gene flow has been reported in other geographically distant canid species (fig. 5*E* and *F*; supplementary table S2, Supplementary Material online), such as between Ethiopian wolf and Eurasian gray wolves and golden jackals, and between Ethiopian wolf and lineage ancestral to northwestern and eastern African wolves (Gopalakrishnan et al. 2018).

**Fig. 6.**
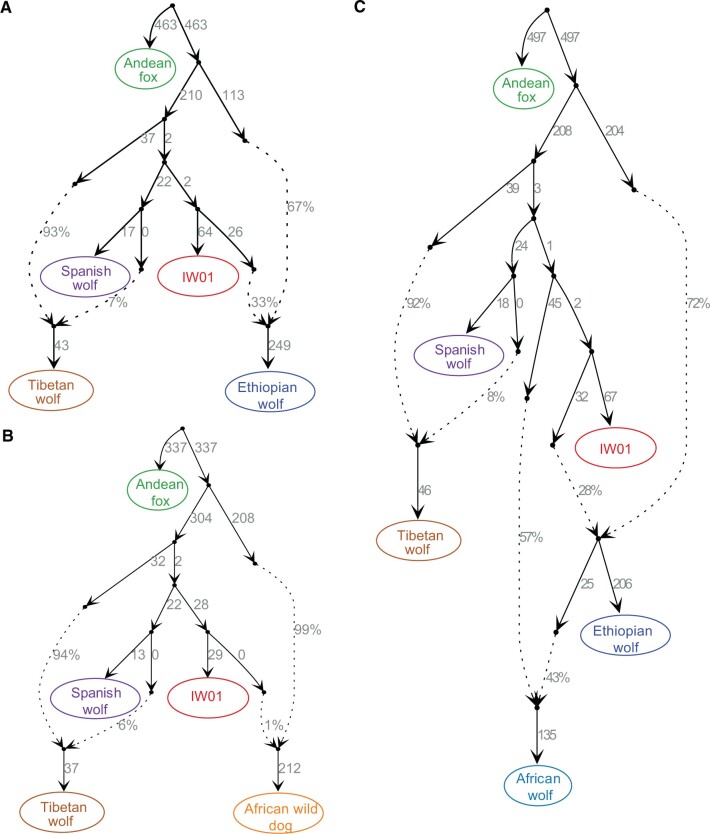
Admixture graph modeling the gene flow between IW01 and African canids. (*A*) Fitted model (no f4 outliers) showing the genetic makeup of the Ethiopian wolf. (*B*) Fitted model (no f4 outliers) showing that the African wild dog carries 1% ancestry from IW01, which supports the *D*-statistics analysis (fig. 5*F*). Admixture between IW01 and African wild dogs is also detected when African wolf or golden jackal are included (supplementary fig. S11, Supplementary Material online). (*C*) Fitted model (no f4 outliers) showing admixture between IW01 and the African wolf and the Ethiopian wolf. This result is also supported by an alternative fitted model (supplementary fig. S11, Supplementary Material online). Both support a previous conclusion that African wolves carry admixed ancestries (Gopalakrishnan et al. 2018). Dashed lines indicate inferred admixture events and admixture proportions are reported beside the dashed lines. Because this analysis required genotype calls, we included only genomes with sequencing coverage >10-fold. As genome sequence coverage for the Himalayan wolf is 7-fold, we used the Tibetan wolf to represent the highland gray wolf for admixture graph construction.

We constructed admixture graph models to further investigate admixture among IW01 and African canids. Because this analysis requires a specified graph topology for testing, it is challenging to implement this test with a large number of populations or species with histories involving complex admixture events. Following previous canid genomic studies (Sinding et al. 2018), we simplified admixture graphs by beginning with a model that includes European wolf, Tibetan wolf, IW01, and Andean fox (as an out-group), and then adding African canid species to fit all possible f4-statistics (Lipson 2020). In agreement with our *D*-statistics results, the fitted admixture graphs (no f4 outliers) indicated that IW01 had gene flow with the African wolf, Ethiopian wolf, and African wild dog (fig. 6 and supplementary fig. S11, Supplementary Material online). We found more gene flow between IW01 and the African wolf and Ethiopian wolf than between IW01 and with the African wild dog. Because the admixture history among gray wolf and canid species is complex, these fitted graphs reflect a parsimonious summary of our data and may not reflect the complete admixture history for these lineages.

Lastly, we applied PCAdmix (Brisbin et al. 2012) to perform local ancestry inference for IW01, with African canids, Eurasian gray wolf, and domestic dog as source populations. Although this analysis has low power and resolution to infer small tracts reflecting anciently admixed ancestry, IW01 shared some potentially admixed tracts (posterior probabilities > 0.9) with each of the three African canid species, the African wolf, Ethiopian wolf, and African wild dog (supplementary table S4, Supplementary Material online). The identified admixed tracts were short and few in number, indicative of ancient gene flow. IW01 shared the largest number and length of admixed blocks with the African wolf, followed by the Ethiopian wolf.

The above analyses support pervasive ancient gene flow between IW01 and African canids. Compared with the two wolves SRS661487 (India) and SRS661488 (Iran), IW01 shares less ancestry with African wolves and a comparable amount of ancestry with the Ethiopian wolf (fig. 5*D* and *E*). A possible explanation for this pattern is that gene flow between IW01 and African canids was mediated through Middle Eastern wolves. However, this model does not explain the shared ancestry between IW01 and African wild dogs (figs. 5*F* and 6; supplementary fig. S11, Supplementary Material online).

Further, our results show that Iranian wolf genomes shared a large excess of genetic ancestry with IW01 (fig. 4 and supplementary fig. S7, Supplementary Material online). This suggests that the lineage leading to IW01 may have been more widely distributed in the past, from the Indian subcontinent to the Arabian Peninsula (Sharma et al. 2004), and overlapping in range and potentially hybridizing with Middle-Eastern gray wolves and African canid lineages in the past.

Our results support the hypothesis that the Sinai Peninsula and Southwest Levant are important hubs of canid evolution, where pervasive interspecific hybridization has been detected among gray wolves, African wolves, and Eurasian golden jackals (Koepfli et al. 2015; Gopalakrishnan et al. 2018). Assemblages of Early Pleistocene mammalian fossils from the Pinjor Formation in India, including remains of at least two species of *Canis*, suggest paleobiogeographic linkages with African and Middle Eastern faunas (Patnaik and Nanda 2010). The connections between the faunas of India and Africa are also supported by the vertebrate fossil records from Late Pleistocene deposits in Gujurat, which includes a *Canis* sp. that is larger and more robust than the present-day Indian wolf (Costa 2017), and from other taxa, as Asiatic lions in India have experienced extensive gene flow with African lions (de Manuel et al. 2020), and African leopards are known to have admixed with leopards from the Middle East (Palestine region) and Central Asia (Afghanistan) (Paijmans et al. 2021). Our model is, of course, speculative, and additional data from both fossils and living animals will be helpful to understand the history of admixture among these canid lineages.

Intriguingly, *D*-statistics tests of allele sharing between IW01 and African canids revealed the Himalayan wolf as distinct from other wolf lineages (fig. 5*C*–*G*), leading us to hypothesize that the Himalayan wolf was less admixed. To test this, we computed *D*-statistics with the Himalayan wolf as H1, domestic dog and gray wolves as H2, and African wolf, Ethiopian wolf, African wild dog, or golden jackal as H3. All analyses resulted in significant positive *D* values (*Z* > 3), suggesting that the domestic dog and gray wolves also shared excess derived alleles with African canids and golden jackals (supplementary fig. S12, Supplementary Material online). This analysis provides support for the idea that present-day wolves and domestic dogs have admixed ancestries (Fan et al. 2016; Frantz et al. 2016) and that the Himalayan wolf is relatively isolated (less or unadmixed with other canids) compared with other wolves (fig. 5*B*).

### Demographic History and Divergence Time for the IW01 and Other Gray Wolves

**Fig. 7.**
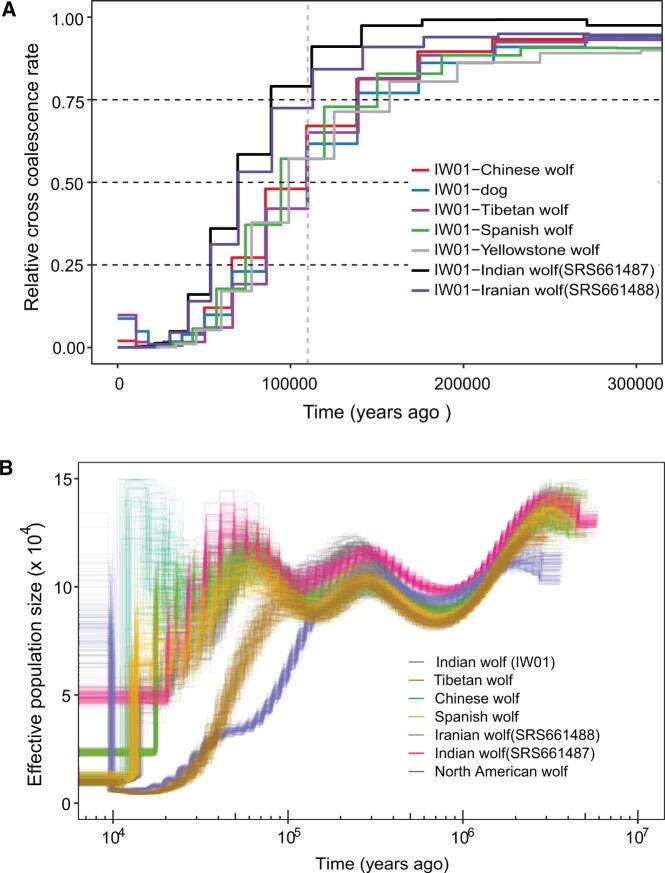
Inferences of the time of divergence and demographic history of Indian wolf IW01 and other gray wolves. (*A*) MSMC estimation of splitting time for Indian wolves (IW01) from the domestic dog and other representative gray wolves. Gray-dashed vertical line indicates the estimated split time at ∼110 ka. (*B*) Results of PSMC analysis showing the demographic trajectories of seven representative gray wolves. For each sample, we performed 100 bootstrap replicates.

To place the evolution of IW01 in a chronological context along with other gray wolves, we calculated relative cross-coalescence rates (CCR, the ratio between the cross- and the within-coalescence rates) for each pair of populations using the multiple sequentially Markovian coalescent (MSMC) model (Schiffels and Durbin 2014), including genomes with a sequence coverage >20-fold. Using 50% CCR as a cutoff to estimate divergence time, these analyses suggest that IW01 diverged from domestic dogs and Chinese, Tibetan, European, and American wolves ∼110 ka (fig. 7*A*). This divergence date is much older than the previous estimates of ∼68–81 ka for divergence between the Tibetan wolf and domestic dog/East Asian gray wolves (Wang et al. 2020) and supports our phylogenetic result that IW01 is basal to the Tibetan/Himalayan wolf and domestic dog + gray wolf clade (figs. 2*C* and 4; supplementary figs. S5–S7, Supplementary Material online). This analysis also showed that IW01 split from SRS661487 (India) and SRS661488 (Iran) more recently, around 86 and 81 ka, respectively (fig. 7*A*), although these estimates will be impacted by the admixed ancestry of these three individuals (fig. 4). We estimated that SRS661487 diverged from the domestic dog and Chinese wolf ∼68–85 ka and from European wolf ∼17 ka (supplementary fig. S13, Supplementary Material online), and that SRS661487 separated from the Iranian wolf (SRS661488) ∼5.5 ka, consistent with these two samples clustering together in the phylogeny (fig. 4 and supplementary figs. S6 and S7, Supplementary Material online). Therefore, SRS661487 likely represents a gray wolf that recently descended from Middle Eastern and European wolf lineages that then admixed with the IW01 lineage, whereas IW01 is a distinct and deeply diverged lineage.

We used the pairwise sequentially Markovian coalescent (PSMC) model (Li and Durbin 2011) to reconstruct historical patterns of effective population size over time for IW01 and other gray wolves with sequencing coverage ≥20-fold (fig. 7*B*). Generally, all gray wolves shared similar demographic trajectories up to ∼150 ka. Thereafter, IW01 and the Tibetan wolf diverged first around 110 ka and then experienced continuous contractions in population size. Generally consistent with MSMC and PSMC, we used Coal-HMM (Mailund et al. 2011) and estimated that IW01 diverged from dogs and other gray wolves ∼130–140 ka (supplementary fig. S14, Supplementary Material online). In contrast, SRS661487 shared a similar demographic trajectory with European, Iranian, and North American wolves whose population size expanded slightly between 100 and 50 ka, which was then followed by contraction (fig. 7*B*). These results corroborate that IW01 and SRS661487 represent two different gray wolf lineages.

To explore the recent history of the Indian wolf population, we examined nucleotide diversity and runs of homozygosity (ROH) for IW01 and compared this with the estimates from other gray wolves. Because such analyses are sensitive to genotyping errors, we focused on genomes with sequencing coverage ≥20-fold. IW01 had a nucleotide diversity of approximately 0.00104 ± 0.00098 (mean±SD), slightly higher than that of the Tibetan wolf, but lower than that estimated for European wolves, SRS661487, the Iranian wolf (SRS661488), the Mongolian wolf, and the North American wolf (supplementary fig. S15, Supplementary Material online). IW01 had 11 blocks of ROH with a length >1 Mb, the longest of which was 1.57 Mb, whereas the Tibetan wolf had 48 blocks of ROH >1 Mb and 5 ROH >2 Mb (supplementary fig. S16, Supplementary Material online). We found that 33% of the IW01 genome and 43% of the Tibetan wolf genome were homozygous, which was higher than that observed in other gray wolves except for the Chinese wolf (supplementary fig. S16, Supplementary Material online). These results are consistent with the long-term small effective population sizes inferred in our PSMC analysis and with earlier ecological studies (Aggarwal et al. 2003, 2007; Sharma et al. 2004), and also suggest recent inbreeding.

## Conclusion

Our results suggest that IW01 represents an evolutionarily distinct gray wolf lineage living in the semi-arid lowland region of the Indian subcontinent that diverged from other gray wolf populations ∼110 ka. IW01 shares ancestry with other gray wolves (SRS661487 and SRS661488) that fall within the geographic range described for *C.l.pallipes*. Consistent with our previous study, gray wolves from the Trans-Himalayan mountain range and Tibetan Plateau also carry deeply diverged ancestries (Wang et al. 2020). The persistence of these ancient and diverged lineages in the Indian subcontinent may be due in part to the region’s unique topography and paleoenvironmental history. Similar patterns of locally divergent lineages have been observed in Trans-Himalayan red pandas (Hu et al. 2020) and Chinese mountain cats (Yu et al. 2021). Together, these findings point to the importance of the Indian subcontinent and Trans-Himalayan region as refugia during the Pleistocene (Sharma et al. 2004; Costa 2017) that enabled the persistence of divergent lineages.

During the Pleistocene ice ages, the Indian subcontinent was dry and cold, and much of the Himalayan and Trans-Himalayan regions and southern Tibet (Owen et al. 2002) were covered by ice. Regional unglaciated refugia persisted, however, within which small populations of gray wolves may have become isolated, leading to the evolution of distinct lineages (Blinkhorn and Petraglia 2017). Our estimate of the timing of divergence between IW01 and other gray wolves coincides roughly with the end of the Last Interglacial period (Eemian), when warmer, wetter conditions occurred in the northern latitudes of Eurasia, whereas the Indian subcontinent and neighboring lower latitude regions experienced a cooler, drier climate (Pedersen et al. 2017). These paleoclimatic differences, combined with geographic isolation, may have facilitated ecological and genetic divergence of the Indian wolf lineage.

Despite the relative isolation and small population size of Indian wolves today, we find that the IW01 lineage harbors evidence of a mosaic of past gene flow with the African wolf, Ethiopian wolf, African wild dog, and western Asian gray wolves. We also find that the Himalayan wolf shares significantly less admixed ancestry with modern-day African canids (supplementary fig. S12, Supplementary Material online), which is consistent with its isolation and adaptation to the high-altitude arid environments of the Himalayan and Tibetan plateaus. It is possible that the distribution of gray wolves and African canids overlapped in the past, possibly in the Sinai Peninsula or Southwest Levant where several canid species are hypothesized to have hybridized (Gopalakrishnan et al. 2018).

Our results present a scenario of pervasive gene flow between gray wolves and other canid species, adding to the growing evidence of the important role of interspecific hybridization in the evolution of canid species and populations specifically and the role of network-linked and reticulated evolution of species more generally. Although our study is based on a single sample of precisely known provenance, our analyses of IW01 bridge a data gap for gray wolves and provide an important resource for future studies. Additional sampling of Indian wolves from other regions of peninsular India, of other wolves from across the range of *C.l.pallipes*, and perhaps from ancient samples will be necessary to inform the conservation of this threatened and elusive gray wolf subspecies.

## Materials and Methods

### IW01: Origins and Sampling

The Indian wolf (IW01; fig. 1 and supplementary fig. S1, Supplementary Material online) sequenced for this study was captured in 1995 inside Velavadar Blackbuck National Park in Gujarat state, India (latitude = 22.0438°N, longitude = 72.0202°E), for a radio-telemetry-based ecological study of the species. The wolf was captured using a rubberized-jaw McBride foot-hold trap (Minnesota) and anesthetized using Telozol (Kreeger et al. 1989). Whole blood was drawn from the brachial vein for DNA profiling and disease study. Permissions for capture and collaring were obtained from the Ministry of Environment and Forest, government of India, and from the Chief Wildlife Warden, Gujarat state. The whole blood sample was stored in alcohol at −20 °C until genomic DNA was extracted.

### Genome Sequencing and Variant Calling

Four paired-end DNA sequencing libraries were prepared for IW01, resulting in a total of 311,789,040 paired-end 150-bp reads (corresponding to 93.5 Gb) generated by the M/s Xcelris Labs Ltd. Ahmedabad, Gujarat, India, using the Illumina HiSeq 2500 platform. We downloaded published genomic sequences from 30 other canid samples from the NCBI SRA (accession IDs are available in supplementary table S1, Supplementary Material online) including domestic dogs, African wild dog (*Lycaon pictus*), dhole (Cuon alpinus), coyote (*Canis latrans*), Eurasian golden jackal (*Canis aureus*), African wolf (*Canis lupaster*), Ethiopian wolf (*Canis simensis*), and Andean fox (*Lycalopex culpaeus*). We used Btrim (Kong 2011) to remove low-quality bases. Because a highly contiguous chromosome-level reference genome assembly is not yet available for the gray wolf, we aligned the remaining reads to the domestic dog CanFam3.1 reference genome (Lindblad-Toh et al. 2005) using the BWA-MEM algorithm (Li 2014) with the settings “-t 4 –M.” We processed the bam alignment by coordinate sorting, marking duplicated reads, performed local realignment, and recalibrated base quality scores using the Picard (version 1.56; http://broadinstitute.github.io/picard/, last accessed January 27, 2022) and GATK (version 3.7.0) packages (McKenna et al. 2010). We called SNPs for all samples together using the UnifiedGenotyper function in GATK. To increase the reliability of the data, SNPs were further filtered as previously described (Wang et al. 2020) using the VariantFiltration command in GATK with parameters: “QUAL < 40.0 MQ < 25.0 MQ0 ≥ 4 && ((MQ0/(1.0×DP)) > 0.1) cluster 3 -window 10.” Index, depth, and mapping statistics were computed using available tools in SAMtools v1.3.1 (Li et al. 2009).

### Mitochondrial Assembly and Phylogenetic Analysis

Because no complete mitochondrial genome is available in GenBank for the Indian wolf, we performed de novo assembly of the mitochondrial genome for IW01, SRS661487 (India), and SRS661488 (Iran) using NOVOPlasty v2.7.2 (Dierckxsens et al. 2017) with a k-mer size of 31 based on whole-genome sequencing data. The domestic dog mitochondrial genome (GenBank accession:NC_002008.4) was used as a seed/reference sequence. We downloaded mitochondrial genomes for coyote, African dog, dhole, African wolf, and other gray wolves and domestic dogs from NCBI (GenBank accessions are shown in fig. 1*C*) and included the Tibetan and Himalayan wolf sequences from a previous study (Wang et al. 2020). A total of 39 mitogenomes were analyzed in this study. These sequences were aligned using MUSCLE v3.8.31 (Edgar 2004) and the alignments were checked manually. After removing poorly aligned and control regions, an alignment file with a length of 15,462 bp was used for phylogenetic analysis. A maximum-likelihood tree was reconstructed using RAxML v8.2.12 (Stamatakis 2014) with the GTR+G model of DNA substitution, and 1,000 bootstraps were run to assess node support.

We also downloaded previously reported mitochondrial cytochrome *b* and 16S rRNA sequences for Indian wolf, domestic dog, and other gray wolves from GenBank (accessions are shown in supplementary fig. S2, Supplementary Material online) and aligned and analyzed these data (554 bp for 16S rRNA and 332 bp for cytochrome *b*) for phylogenetic analysis using the same methods described above.

### Nuclear Phylogeny Construction

We constructed phylogenetic trees using nuclear genome sequences to explore the relationship of IW01 with other gray wolves and canid species. For each canid taxon, only one sample was used. Given that domestic dogs constitute a monophyletic clade (Fan et al. 2016; Wang et al. 2016), we chose the high-coverage Dingo genome (31.3-fold; SRR7120191) to represent the domestic dog lineage. As a result, a total of 19 samples were used to construct phylogenetic trees (fig. 2*A* and supplementary fig. S3, Supplementary Material online). We generated a consensus genome for each sample using ANGSD v0.931 (Korneliussen et al. 2014) (-doFasta 1). Reads with a minimum mapping quality lower than 25 were discarded (-minMapQ 25). For genomes with an average sequencing depth of over or less than 10-fold, the minimum depth for each base was set to 4-fold (-setMinDepth 4) or to 3-fold (-setMinDepth 3), respectively. Additional filter parameters implemented were: -doCounts 1 -uniqueOnly 1 -nThreads 2. We selected 5,000 random regions with a length of 20 kb from across the genome of the domestic dog reference assembly and the other 18 canid taxa using the “random” function in BEDTools v2.28.0 (Quinlan and Hall 2010) (-l 20,000 -n 5,000). Sequences for each region were retrieved using the “faidx” function of SAMtools v1.3.1 (Li et al. 2009). For each region, a maximum-likelihood tree was constructed by RAxML v8.2.12 (Stamatakis 2014) with 100 bootstrap replicates using the command: raxmlHPC-PTHREADS-SSE3 -x 12,345 -k -# 100 -p 321 -m GTRGAMMAI -T 4 -s myseq.fas -f a -n myseq.ml.tre. The 5,000 gene trees were then concatenated and used as input for ASTRAL-III v5.7.5 (Zhang et al. 2018) to generate a species tree, using default parameters. We used DiscoVista (Sayyari et al. 2018) to analyze the discordance frequencies between the ASTRAL species tree and the 5,000 gene trees.

We retrieved and concatenated genotypes for 31 samples (in VCF format) within regions containing the signal of diverged origin in high-altitude wolves (Himalayan and Tibetan wolves) (Wang et al. 2020), and converted into .fas format files. A neighbor-joining tree was constructed using the mega-cc tool (Kumar et al. 2012) in MEGA7 (Kumar et al. 2016) and nodal support was evaluated with 1,000 bootstrap replicates. Lastly, following (Wang et al. 2020), we split four high-coverage genomes from the Chinese wolf, IW01, Tibetan wolf, and dhole into 250-, 500-, and 1,000-kb windows across autosomes and constructed phylogenies for each window using TreeMix v1.13 (Pickrell and Pritchard 2012) with dhole as the outgroup. The frequency of each topology was calculated using APE v5.5 (Popescu et al. 2012).

### PSMC Analysis

We used the PSMC model to infer historical demographic trajectories for the sampled gray wolves (Li and Durbin 2011). We only analyzed genomes with coverage >20-fold to ensure the accurate calling of heterozygotes (Nadachowska-Brzyska et al. 2016), although some studies used low coverage genomes with false negative rate corrections (Kim et al. 2014; Hawkins et al. 2018). A diploid consensus sequence for each individual was generated using the “mpileup” command of the SAMtools package (v1.3.1) (Li et al. 2009) with the option “-C50.” Variants with less than about 1/3 (“-d” option) or over two times (“-D” option) of average read depth were marked as missing and excluded from consensus sequence assignment. Sequences with consensus quality lower than 20 were also filtered out. The program “fq2psmcfa” from the PSMC package was used to convert the consensus sequences into 100-bp bin-input files for PSMC. We ran PSMC with parameters “-N25 -t15 -r5 -p 4 + 25×2 + 4 + 6.” A total of 100 bootstraps were analyzed for each sample. These PSMC estimates are scaled using a generation time (*g*) of 3 years and a mutation rate (µ) of 4e − 9 substitutions per site per generation as used previously (Skoglund et al. 2015). This mutation rate was comparable to a recent estimation based on pedigree analysis (Koch et al. 2019).

### MSMC and Coal-HMM Inference of Splitting Time

We used the multiple sequential Markovian coalescent (MSMC2) model to infer the divergence time for the domestic dog and gray wolf population pairs (Schiffels and Durbin 2014). Genotypes for all dogs and wolves were phased together using Beagle V.4.1 (Browning and Browning 2016). The MSMC input files comprising four haplotypes (two individuals) were generated as suggested by the authors using available tools from the MSMC-tool package (https://github.com/stschiff/msmc-tools, last accessed January 27, 2022). We ran MSMC for each pair of genomes using default settings and the time when the relative cross-coalescent rate was dropped to 50% as an approximate estimate of the splitting time (Malaspinas et al. 2016). For each calculation, four haplotypes were analyzed, and estimations were scaled using a generation time (*g*) of 3 years and a mutation rate (µ) of 4e −9 substitutions per site per generation (Skoglund et al. 2015). Similar to the PSMC analysis, we restricted this analysis to genomes with coverage >20-fold.

We also used Coal-HMM (Mailund et al. 2011), a coalescent hidden Markov model-based approach, to measure the divergence time for the Indian wolf (IW01) and dog and other wolves. We performed estimation for each population pair using 1-Mb nonoverlapping sliding window segments across each chromosome. We filtered out windows with over 10% missing rate for such analysis. We also removed results for each segment where: 1) the recombination rate was lower 0.1 or over 10 cM/Mb, 2) the ancestral effective population size below 1,000 or above 1,000,000, and 3) the split time was below 1,000 years or above 1,000,000 years.

### Nuclear Diversity and ROH Analysis

Nucleotide diversity (π) (Nei and Li 1979) was calculated for each sample across the autosomes using VCFtools v0.1.13 (Danecek et al. 2011) in 50-kb sliding windows with a step of 25 kb. ROH was calculated for each sample across the autosomes using the “roh” function in the BCFtools v1.4-7-g41827a3 (Narasimhan et al. 2016) with default parameters.

### TreeMix, ABBA-BABA, and AdmixtureGraph Analyses

To explore the phylogenetic relationships and admixture among gray wolves and other canid species, we also used TreeMix v1.13 (Pickrell and Pritchard 2012) to construct maximum-likelihood tree graphs by allowing gene flow. TreeMix analysis was run for all variants located on autosomes using 1,000 variants per block (-k 1,000) and allowing zero to five migrations, with Andean fox used as the outgroup.

We used the ABBA-BABA test, also known as *D*-statistics (Green et al. 2010) to detect the amount of allele sharing between gray wolf populations. This analysis is based on the topology (((H1, H2), H3), Outgroup) as shown in figure 5*A*. *D* = 0 suggests no gene flow between ingroup (H1 or H2) and H3; *D* > 0 suggests gene flow between H3 and H2; and *D* < 0 suggests gene flow between H3 and H1. We used the function “-doAbbababa 1” in ANGSD v0.931 (Korneliussen et al. 2014) to perform this analysis with the additional settings “-doCounts 1 -minMapQ 25 -minQ 25 -uniqueOnly 1 -nThreads 6.”

To assess the genetic makeup and relationships among IW01, gray wolves, and three African canid species (African wolf, Ethiopian wolf, and African wild dog), we constructed admixture graph models using the *qpGraph* tool from AdmixTools package (Patterson et al. 2012), the admixturegraph R package (Leppala et al. 2017), and qpBrute (Ni Leathlobhair et al. 2018; Liu et al. 2019). Because this analysis requires high-confidence genotype calls, we chose one sample with genome sequencing coverage over 10-fold from each population or species for constructing admixture graphs. To resolve the relationship between IW01, Himalayan/Tibetan wolves, and Eurasian gray wolves, we tested all possible graph models to fit all possible f4-statistics. The phylogenetic tree based on “ghost” admixed sequences and mitochondrial genomes from Himalayan or Tibetan wolves showed that the “ghost” lineage was basal to IW01. Therefore, we considered graphs in which Himalayan or Tibetan wolves were modeled as a product of admixture with one source from the lineage basal to IW01. To investigate the admixture between IW01 and African canids, we constructed admixture models starting with three populations (IW01, European wolf, and Himalayan or Tibetan wolf) and the fitted graph was then used as the base model in which we successively added each of the three African canid species.

### Local Ancestry Inference

To identify potential admixed tracts along each chromosome in IW01, we performed local ancestry inference using PCAdmix (Brisbin et al. 2012). We used phased genotypes as mentioned above as input, with IW01 designated as an admixed population and each of the African canid species, domestic dog, and Eurasian gray wolves as source populations. We performed two independent runs using 20 (default by the software) and 40 SNPs per window (“-w” parameter), respectively. The identified regions with posterior probabilities >0.9 were considered as potentially admixed.

## Supplementary Material

Supplementary data are available at *Genome Biology and Evolution* online.

## Supplementary Material

evac012_Supplementary_Data

## References

[evac012-B1] Aggarwal RK , Kivisild T, Ramadevi J, Singh L. 2007. Mitochondrial DNA coding region sequences support the phylogenetic distinction of two Indian wolf species. J Zool Syst. 45(2):163–172.

[evac012-B2] Aggarwal RK , Ramadevi J, Singh L. 2003. Ancient origin and evolution of the Indian wolf: evidence from mitochondrial DNA typing of wolves from trans-Himalayan region and Pennisular India. Genome Biol. 4(6):P6.

[evac012-B3] Allen GM. 1938. The mammals of China and Mongolia. Natural history of Central Asia. XI, Part I. 1st ed. New York: The American Museum of Natural History. p.342–345.

[evac012-B4] Allman ES , Degnan JH, Rhodes JA. 2011. Identifying the rooted species tree from the distribution of unrooted gene trees under the coalescent. J Math Biol. 62(6):833–862. 20652704 10.1007/s00285-010-0355-7

[evac012-B5] Amiri Ghanatsaman Z , et al 2020. Whole genome resequencing of the Iranian native dogs and wolves to unravel variome during dog domestication. BMC Genomics 21(1):207. 32131720 10.1186/s12864-020-6619-8 PMC7057629

[evac012-B6] Blinkhorn J , Petraglia MD. 2017. Environments and cultural change in the Indian Subcontinent: implications for the dispersal of *Homo sapiens* in the Late Pleistocene. Curr Anthropol. 58(S17):S463–S479.

[evac012-B7] Brisbin A , et al 2012. PCAdmix: principal components-based assignment of ancestry along each chromosome in individuals with admixed ancestry from two or more populations. Hum Biol. 84(4):343–364. 23249312 10.3378/027.084.0401 PMC3740525

[evac012-B8] Browning BL , Browning SR. 2016. Genotype imputation with millions of reference samples. Am J Hum Genet. 98(1):116–126. 26748515 10.1016/j.ajhg.2015.11.020 PMC4716681

[evac012-B9] Chavez DE , et al 2019. Comparative genomics provides new insights into the remarkable adaptations of the African wild dog (*Lycaon pictus*). Sci Rep. 9(1):8329. 31171819 10.1038/s41598-019-44772-5 PMC6554312

[evac012-B10] Costa AG. 2017. A new Late Pleistocene fauna from arid coastal India: implications for inundated coastal refugia and human dispersals. Quat Int. 436:253–269.

[evac012-B11] Danecek P , et al 2011. The variant call format and VCFtools. Bioinformatics 27(15):2156–2158. 21653522 10.1093/bioinformatics/btr330 PMC3137218

[evac012-B12] Degnan JH , Rosenberg NA. 2009. Gene tree discordance, phylogenetic inference and the multispecies coalescent. Trends Ecol Evol. 24(6):332–340. 19307040 10.1016/j.tree.2009.01.009

[evac012-B13] de Manuel M , et al 2020. The evolutionary history of extinct and living lions. Proc Natl Acad Sci U S A. 117:10927–10934. 32366643 10.1073/pnas.1919423117 PMC7245068

[evac012-B14] Dierckxsens N , Mardulyn P, Smits G. 2017. NOVOPlasty: de novo assembly of organelle genomes from whole genome data. Nucleic Acids Res. 45(4):e18. 28204566 10.1093/nar/gkw955 PMC5389512

[evac012-B15] Edgar RC. 2004. MUSCLE: multiple sequence alignment with high accuracy and high throughput. Nucleic Acids Res. 32(5):1792–1797. 15034147 10.1093/nar/gkh340 PMC390337

[evac012-B16] Ersmark E , et al 2016. From the past to the present: wolf phylogeography and demographic history based on the mitochondrial control region. Front Ecol Evol. 4. doi: 10.3389/fevo.2016.00134.

[evac012-B17] Fan Z , et al 2016. Worldwide patterns of genomic variation and admixture in gray wolves. Genome Res. 26(2):163–173. 26680994 10.1101/gr.197517.115 PMC4728369

[evac012-B18] Frantz LA , et al 2016. Genomic and archaeological evidence suggest a dual origin of domestic dogs. Science 352(6290):1228–1231. 27257259 10.1126/science.aaf3161

[evac012-B19] Freedman AH , et al 2014. Genome sequencing highlights the dynamic early history of dogs. PLoS Genet. 10(1):e1004016. 24453982 10.1371/journal.pgen.1004016 PMC3894170

[evac012-B20] Gopalakrishnan S , et al 2018. Interspecific gene flow shaped the evolution of the genus *Canis*. Curr Biol. 28(21):3441–3449.e3445. 30344120 10.1016/j.cub.2018.08.041 PMC6224481

[evac012-B21] Green RE , et al 2010. A draft sequence of the Neandertal genome. Science 328(5979):710–722. 20448178 10.1126/science.1188021 PMC5100745

[evac012-B22] Hawkins MTR , et al 2018. Genome sequence and population declines in the critically endangered greater bamboo lemur (*Prolemur simus*) and implications for conservation. BMC Genomics 19(1):445. 29884119 10.1186/s12864-018-4841-4 PMC5994045

[evac012-B23] Hodgson B. 1847. Description of the wild ass and wolf of Tibet. Calcutta J Nat Hist. 7:469–477.

[evac012-B24] Hu Y , et al 2020. Genomic evidence for two phylogenetic species and long-term population bottlenecks in red pandas. Sci Adv. 6(9):eaax5751. 32133395 10.1126/sciadv.aax5751 PMC7043915

[evac012-B25] Jhala Y. 2003. Status, ecology and conservation of the Indian wolf *Canis lupus pallipes* sykes. J Bombay Nat Hist Soc. 100:293–307.

[evac012-B26] Kim HL , et al 2014. Khoisan hunter-gatherers have been the largest population throughout most of modern-human demographic history. Nat Commun. 5:5692. 25471224 10.1038/ncomms6692 PMC4268704

[evac012-B27] Koch E , et al 2019. De novo mutation rate estimation in wolves of known pedigree. Mol Biol Evol. 36(11):2536–2547. 10.1093/molbev/msz159 PMC6805234 31297530

[evac012-B28] Koepfli KP , et al 2015. Genome-wide evidence reveals that African and Eurasian golden jackals are distinct species. Curr Biol. 25(16):2158–2165. 26234211 10.1016/j.cub.2015.06.060

[evac012-B29] Kong Y. 2011. Btrim: a fast, lightweight adapter and quality trimming program for next-generation sequencing technologies. Genomics 98(2):152–153. 21651976 10.1016/j.ygeno.2011.05.009

[evac012-B30] Korneliussen TS , Albrechtsen A, Nielsen R. 2014. ANGSD: analysis of next generation sequencing data. BMC Bioinformatics 15:356. 25420514 10.1186/s12859-014-0356-4 PMC4248462

[evac012-B31] Kreeger TJ , Mandsager RE, Seal US, Callahan M, Beckel M. 1989. Physiological response of gray wolves to butorphanol-xylazine immobilization and antagonism by naloxone and yohimbine. J Wildl Dis. 25(1):89–94. 2915407 10.7589/0090-3558-25.1.89

[evac012-B32] Kumar S , Stecher G, Peterson D, Tamura K. 2012. MEGA-CC: computing core of molecular evolutionary genetics analysis program for automated and iterative data analysis. Bioinformatics 28(20):2685–2686. 22923298 10.1093/bioinformatics/bts507 PMC3467750

[evac012-B33] Kumar S , Stecher G, Tamura K. 2016. MEGA7: molecular evolutionary genetics analysis version 7.0 for bigger datasets. Mol Biol Evol. 33(7):1870–1874. 27004904 10.1093/molbev/msw054 PMC8210823

[evac012-B34] Leonard JA , et al 2007. Megafaunal extinctions and the disappearance of a specialized wolf ecomorph. Curr Biol. 17(13):1146–1150. 17583509 10.1016/j.cub.2007.05.072

[evac012-B35] Leppala K , Nielsen SV, Mailund T. 2017. admixturegraph: an R package for admixture graph manipulation and fitting. Bioinformatics 33(11):1738–1740. 28158333 10.1093/bioinformatics/btx048 PMC5447235

[evac012-B36] Li H. 2014. Toward better understanding of artifacts in variant calling from high-coverage samples. Bioinformatics 30(20):2843–2851. 24974202 10.1093/bioinformatics/btu356 PMC4271055

[evac012-B37] Li H , Durbin R. 2011. Inference of human population history from individual whole-genome sequences. Nature 475(7357):493–496. 21753753 10.1038/nature10231 PMC3154645

[evac012-B38] Li H , et al 2009. The Sequence Alignment/Map format and SAMtools. Bioinformatics 25(16):2078–2079. 19505943 10.1093/bioinformatics/btp352 PMC2723002

[evac012-B39] Lindblad-Toh K , et al 2005. Genome sequence, comparative analysis and haplotype structure of the domestic dog. Nature 438(7069):803–819. 16341006 10.1038/nature04338

[evac012-B40] Lipson M. 2020. Applying f4-statistics and admixture graphs: theory and examples. Mol Ecol Resour. 20(6):1658–1667. 32717097 10.1111/1755-0998.13230 PMC11563031

[evac012-B41] Liu L , et al 2019. Genomic analysis on pygmy hog reveals extensive interbreeding during wild boar expansion. Nat Commun. 10:1992. 31040280 10.1038/s41467-019-10017-2 PMC6491599

[evac012-B42] Liu Y-H , et al 2018. Whole-genome sequencing of African dogs provides insights into adaptations against tropical parasites. Mol Biol Evol. 35(2):287–298. 29040727 10.1093/molbev/msx258

[evac012-B43] Loog L , et al 2020. Ancient DNA suggests modern wolves trace their origin to a late Pleistocene expansion from Beringia. Mol Ecol. 29(9):1596–1610. 31840921 10.1111/mec.15329 PMC7317801

[evac012-B44] Mailund T , Dutheil JY, Hobolth A, Lunter G, Schierup MH. 2011. Estimating divergence time and ancestral effective population size of Bornean and Sumatran orangutan subspecies using a coalescent hidden Markov model. PLoS Genet. 7(3):e1001319. 21408205 10.1371/journal.pgen.1001319 PMC3048369

[evac012-B45] Malaspinas AS , et al 2016. A genomic history of Aboriginal Australia. Nature 538(7624):207–214. 27654914 10.1038/nature18299 PMC7617037

[evac012-B46] McKenna A , et al 2010. The Genome Analysis Toolkit: a MapReduce framework for analyzing next-generation DNA sequencing data. Genome Res. 20(9):1297–1303. 20644199 10.1101/gr.107524.110 PMC2928508

[evac012-B47] Nadachowska-Brzyska K , Burri R, Smeds L, Ellegren H. 2016. PSMC analysis of effective population sizes in molecular ecology and its application to black-and-white Ficedula flycatchers. Mol Ecol. 25(5):1058–1072. 26797914 10.1111/mec.13540 PMC4793928

[evac012-B48] Narasimhan V , et al 2016. BCFtools/RoH: a hidden Markov model approach for detecting autozygosity from next-generation sequencing data. Bioinformatics 32(11):1749–1751. 26826718 10.1093/bioinformatics/btw044 PMC4892413

[evac012-B49] Nei M , Li WH. 1979. Mathematical model for studying genetic variation in terms of restriction endonucleases. Proc Natl Acad Sci U S A. 76(10):5269–5273. 291943 10.1073/pnas.76.10.5269 PMC413122

[evac012-B50] Ni Leathlobhair M , et al 2018. The evolutionary history of dogs in the Americas. Science 361(6397):81–85. 29976825 10.1126/science.aao4776 PMC7116273

[evac012-B51] Nowak RM. 1979. North American quaternary Canis. Lawrence (KS): Museum of Natural History, University of Kansas.

[evac012-B52] Nowak RM. 1995. Another look at wolf taxonomy. In: Carbyn LN, Fritts SH, Seip DR, editors. Ecology and conservation of wolves in a changing world. Edmonton (AB): Canadian Circumpolar Institute. p. 375–398.

[evac012-B53] Owen LA , Finkel RC, Caffee MW. 2002. A note on the extent of glaciation throughout the Himalaya during the global Last Glacial Maximum. Quat Sci Rev. 21(1–3):147–157.

[evac012-B54] Paijmans JLA , et al 2021. African and Asian leopards are highly differentiated at the genomic level. Curr Biol. 31(9):1872–1882. 33848458 10.1016/j.cub.2021.03.084

[evac012-B55] Patnaik R , Nanda AC. 2010. Early Pleistocene mammalian faunas of India and evidence of connections with other parts of the world. In: Fleagle JG, Shea JJ, Grine FE, Baden AL, Leakey RE, editors. Out of Africa I. Dordrecht (The Netherlands): Springer Netherlands. p. 129–143.

[evac012-B56] Patterson N , et al 2012. Ancient admixture in human history. Genetics 192(3):1065–1093. 22960212 10.1534/genetics.112.145037 PMC3522152

[evac012-B57] Pedersen RA , Langen PL, Vinther BM. 2017. The last interglacial climate: comparing direct and indirect impacts of insolation changes. Clim Dyn. 48(9–10):3391–3407.

[evac012-B58] Perri AR , et al 2021. Dire wolves were the last of an ancient New World canid lineage. Nature 591(7848):87–91. 33442059 10.1038/s41586-020-03082-x

[evac012-B59] Pickrell JK , Pritchard JK. 2012. Inference of population splits and mixtures from genome-wide allele frequency data. PLoS Genet. 8(11):e1002967. 23166502 10.1371/journal.pgen.1002967 PMC3499260

[evac012-B60] Pilot M , et al 2019. Global phylogeographic and admixture patterns in grey wolves and genetic legacy of an ancient Siberian lineage. Sci Rep. 9(1):17328. 31757998 10.1038/s41598-019-53492-9 PMC6874602

[evac012-B61] Popescu AA , Huber KT, Paradis E. 2012. ape 3.0: new tools for distance-based phylogenetics and evolutionary analysis in R. Bioinformatics 28(11):1536–1537. 22495750 10.1093/bioinformatics/bts184

[evac012-B62] Quinlan AR , Hall IM. 2010. BEDTools: a flexible suite of utilities for comparing genomic features. Bioinformatics 26(6):841–842. 20110278 10.1093/bioinformatics/btq033 PMC2832824

[evac012-B63] Sayyari E , Whitfield JB, Mirarab S. 2018. DiscoVista: interpretable visualizations of gene tree discordance. Mol Phylogenet Evol. 122:110–115. 29421312 10.1016/j.ympev.2018.01.019

[evac012-B64] Schiffels S , Durbin R. 2014. Inferring human population size and separation history from multiple genome sequences. Nat Genet. 46(8):919–925. 24952747 10.1038/ng.3015 PMC4116295

[evac012-B65] Sharma DK , Maldonado JE, Jhala YV, Fleischer RC. 2004. Ancient wolf lineages in India. Proc Biol Sci. 271(Suppl 3):S1–S4. 15101402 10.1098/rsbl.2003.0071 PMC1809981

[evac012-B66] Sinding MS , et al 2018. Population genomics of grey wolves and wolf-like canids in North America. PLoS Genet. 14(11):e1007745. 30419012 10.1371/journal.pgen.1007745 PMC6231604

[evac012-B67] Skoglund P , Ersmark E, Palkopoulou E, Dalen L. 2015. Ancient wolf genome reveals an early divergence of domestic dog ancestors and admixture into high-latitude breeds. Curr Biol. 25(11):1515–1519. 26004765 10.1016/j.cub.2015.04.019

[evac012-B68] Smeds L , Kojola I, Ellegren H. 2019. The evolutionary history of grey wolf Y chromosomes. Mol Ecol. 28(9):2173–2191. 30788868 10.1111/mec.15054 PMC6850511

[evac012-B69] Stamatakis A. 2014. RAxML version 8: a tool for phylogenetic analysis and post-analysis of large phylogenies. Bioinformatics 30(9):1312–1313. 24451623 10.1093/bioinformatics/btu033 PMC3998144

[evac012-B70] Sykes WH. 1831. Catalogue of the Mammalia of Dukun (Deccan); with observations on the habits, etc., and characters of new species. Proc Zool Soc Lond. 1830–1831:99–106.

[evac012-B71] Thalmann O , et al 2013. Complete mitochondrial genomes of ancient canids suggest a European origin of domestic dogs. Science 342(6160):871–874. 24233726 10.1126/science.1243650

[evac012-B72] Toews DP , Brelsford A. 2012. The biogeography of mitochondrial and nuclear discordance in animals. Mol Ecol. 21(16):3907–3930. 22738314 10.1111/j.1365-294X.2012.05664.x

[evac012-B73] vonHoldt BM , et al 2016. Whole-genome sequence analysis shows that two endemic species of North American wolf are admixtures of the coyote and gray wolf. Sci Adv. 2(7):e1501714. 29713682 10.1126/sciadv.1501714 PMC5919777

[evac012-B74] Wang GD , et al 2016. Out of southern East Asia: the natural history of domestic dogs across the world. Cell Res. 26(1):21–33. 26667385 10.1038/cr.2015.147 PMC4816135

[evac012-B75] Wang MS , et al 2020. Ancient hybridization with an unknown population facilitated high-altitude adaptation of canids. Mol Biol Evol. 37(9):2616–2629. 32384152 10.1093/molbev/msaa113

[evac012-B76] Werhahn G , et al 2018. The unique genetic adaptation of the Himalayan wolf to high-altitudes and consequences for conservation. Glob Ecol Conserv. 16:e00455.

[evac012-B77] Werhahn G , et al 2017. Phylogenetic evidence for the ancient Himalayan wolf: towards a clarification of its taxonomic status based on genetic sampling from western. R Soc Open Sci. 4(6):170186. 28680672 10.1098/rsos.170186 PMC5493914

[evac012-B78] Wilson DE , Reeder DM. 2005. Mammal species of the world: a taxonomic and geographic reference. Baltimore (MD): Johns Hopkins University Press.

[evac012-B79] Yu H , et al 2021. Genomic evidence for the Chinese mountain cat as a wildcat conspecific (*Felis silvestris bieti*) and its introgression to domestic cats. Sci Adv. 7:eabg0221. 34162544 10.1126/sciadv.abg0221 PMC8221621

[evac012-B80] Zhang C , Rabiee M, Sayyari E, Mirarab S. 2018. ASTRAL-III: polynomial time species tree reconstruction from partially resolved gene trees. BMC Bioinformatics 19(S6):153. 29745866 10.1186/s12859-018-2129-y PMC5998893

