# Supplementary material for: Genome Sequencing of a Gray Wolf from Peninsular India Provides New Insights into the Evolution and Hybridization of Gray Wolves
Source: Genome Biol Evol. 2022 Feb 8;14(2):evac012. doi: 10.1093/gbe/evac012 (PMC8841465; doi:10.1093/gbe/evac012)
Supplement: evac012_Supplementary_Data [file evac012_supplementary_data.doc]

**Supplemental Information**

**Genome sequencing a gray wolf from peninsular India provides new insights into the evolution and hybridization of gray wolves**

Ming-Shan Wang, Mukesh Thakur, Yadvendradev Jhala, Sheng Wang, Yellapu Srinivas, Shan-Shan Dai, Zheng-Xi Liu, Hong-Man Cheng, Richard E. Green, Klaus-Peter Koepfli, Beth Shapiro


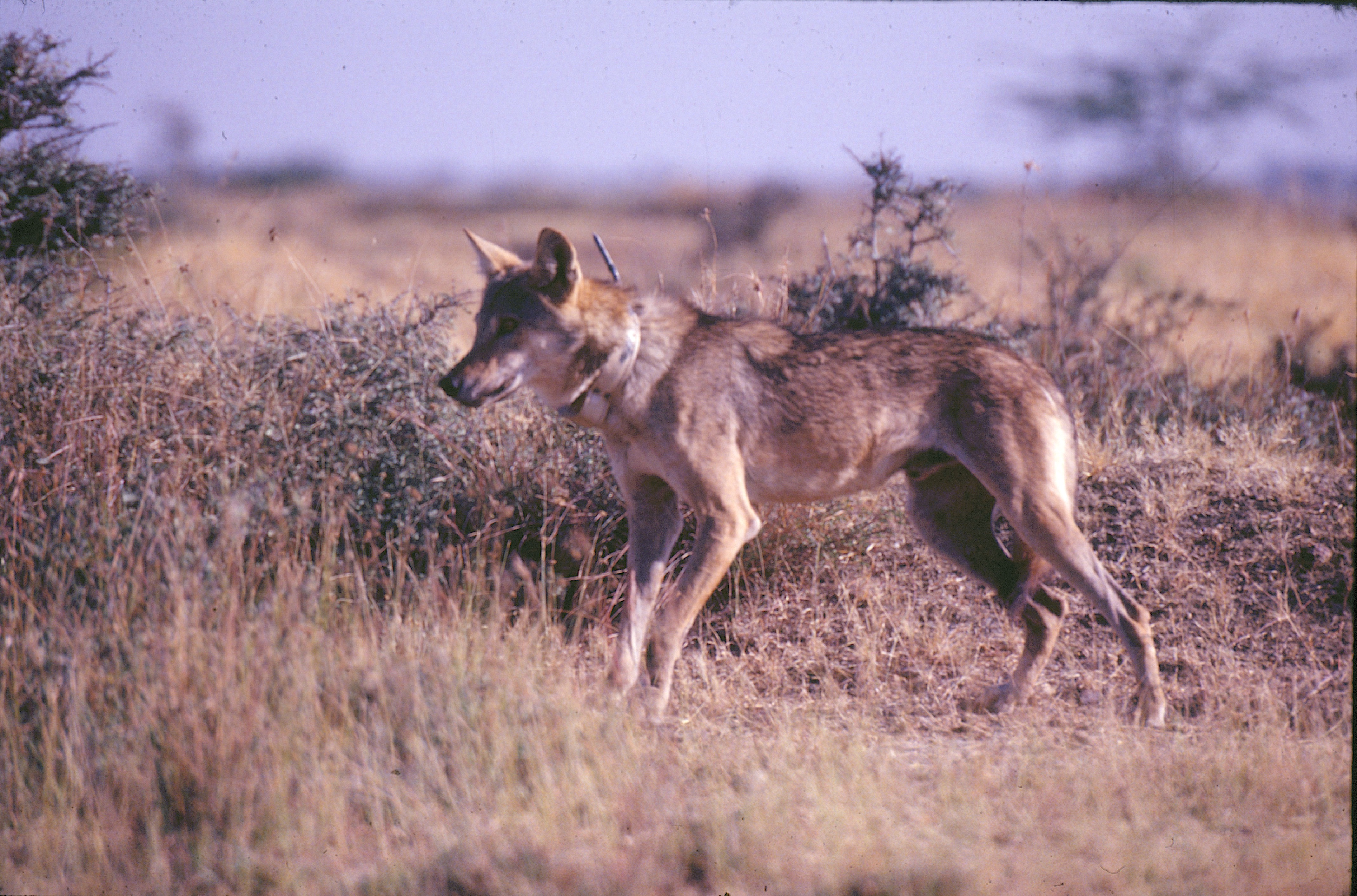


Figure S1: Photo of the Indian wolf (IW01) from Velavadar Blackbuck National Park, Gujarat State, India (latitude = 22.0438º N, longitude = 72.0202º E) sequenced for this study (provided by Yadvendradev Jhala).


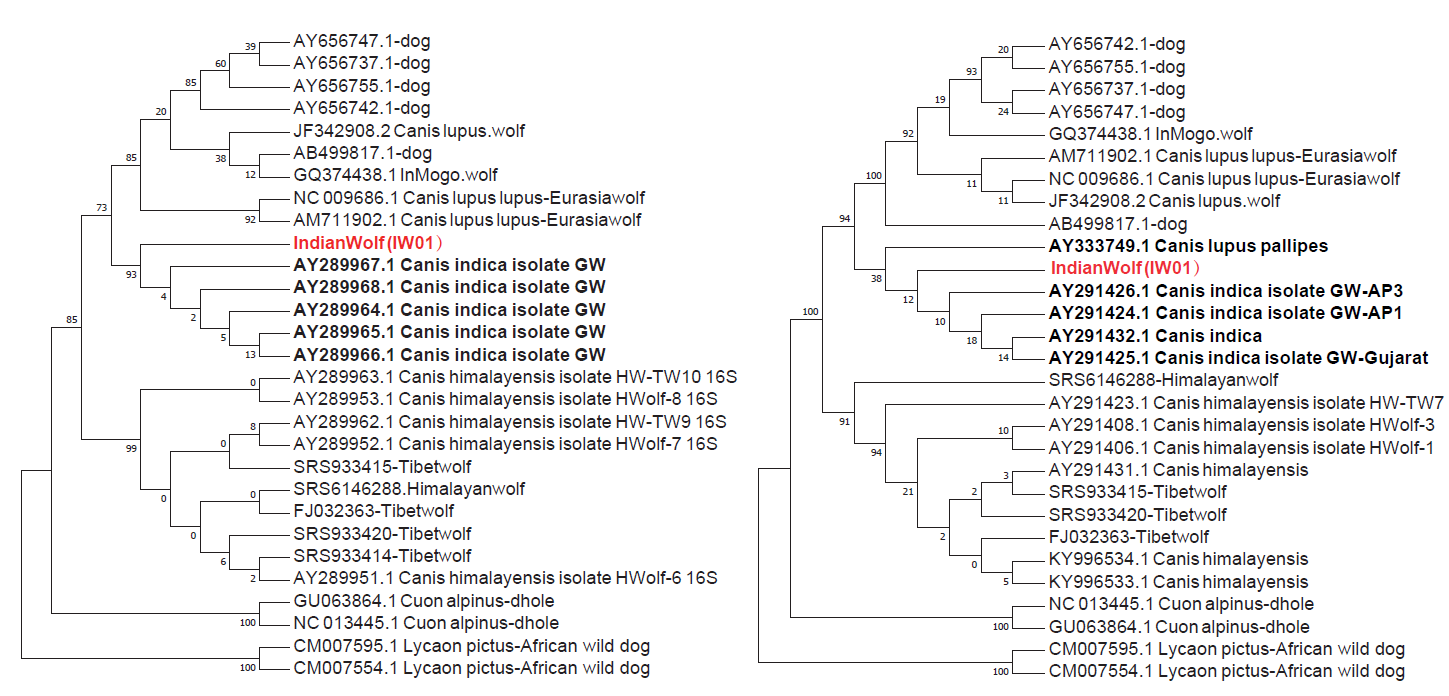


Figure S2: Maximum-likelihood trees for IW01 and other gray wolves based on 16S rRNA (left; 554bp) and cytochrome *b* (right; 332 bp) sequences. IW01 (in red) groups with previously reported Indian wolves (in bold) as a single clade sister to Holarctic gray wolves and domestic dogs. GenBank accession numbers are shown in each lineage. The trees based on the two genes indicate that IW01 groups with the previously reported sequences for Indian wolves ^21,24,25^, holding a deep and distinct position sister to the domestic dog and other modern gray wolves but splitting subsequent to the lineage containing Himalayan and Tibetan wolves.


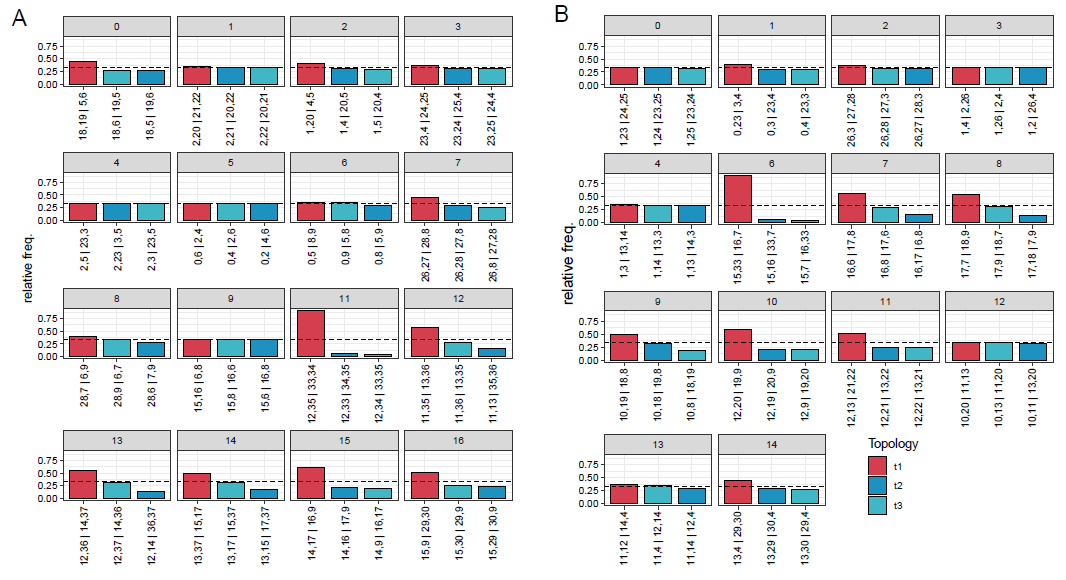


Figure S3: The quartet frequencies of three topologies around each branch at 16 nodes in the underlying unrooted phylogeny as shown in Figure 2A (A) and Figure 2C (B).


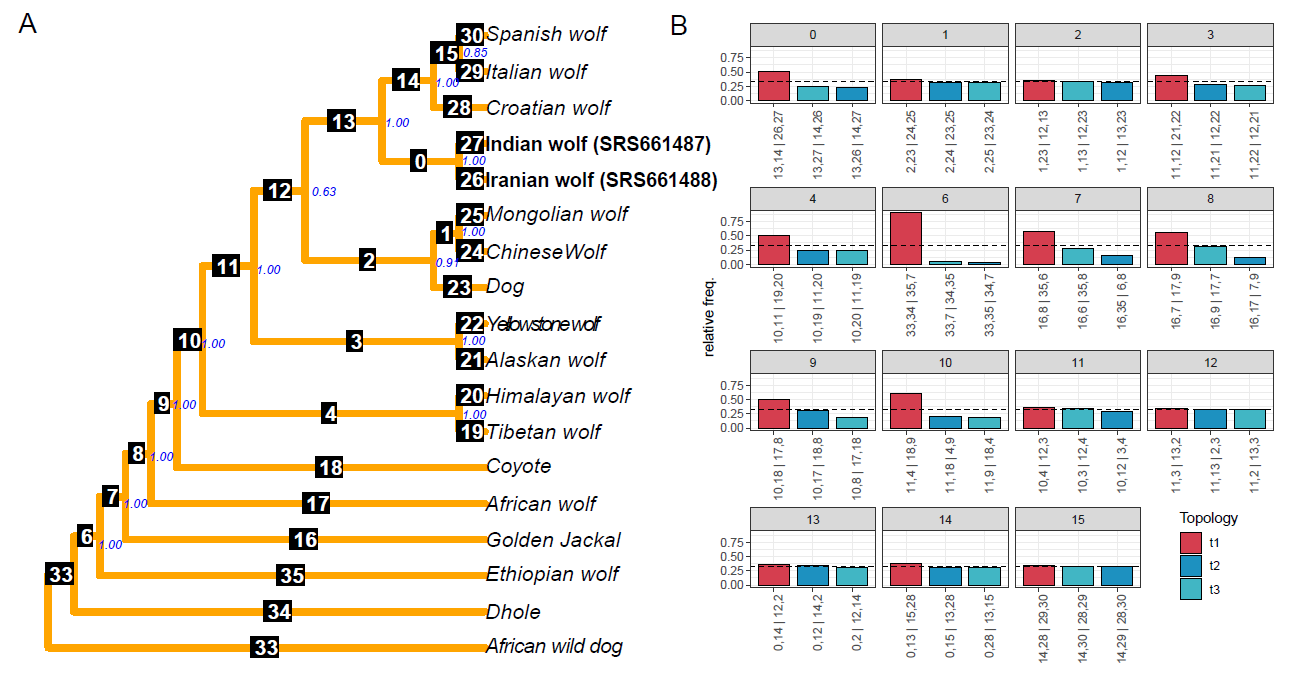


Figure S4: (A) ASTRAL-III tree estimated from the nuclear genome by excluding IW01. (B) The quartet frequencies of three topologies around each branch at 15 nodes in the underlying unrooted phylogeny as shown in Figure S3A.


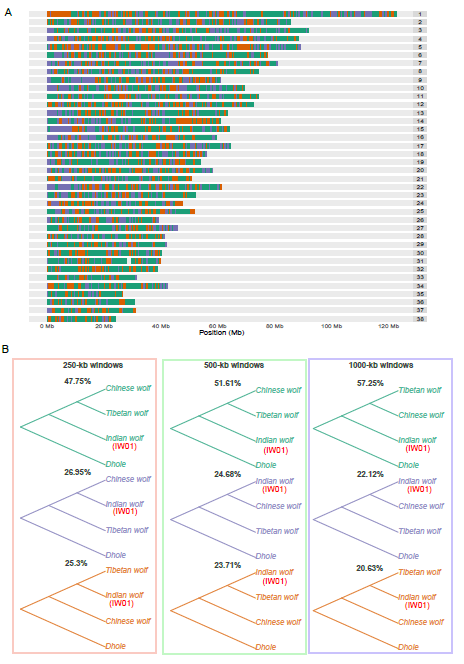


Figure S5: (A) An example showing the distributions of constructed tree topologies using 500kb windows across each autosome. (B) This plot depicts the proportion of three major topologies estimated with 250kb, 500kb, and 1Mb segments. The proportion of the top three most frequent topologies for each window size are shown in each colored box. The topology showing that IW01 and Chinese wolf clustered as a clade sister to Tibetan wolf (22-27% of windows) is more frequent than that showing the Chinese wolf is basal to IW01-Tibetan wolf clade (20.6-25.3%), supporting our previous study that high-altitude gray wolves carried ancestries from a deeply diverged ghost lineage (Wang, et al. 2020).


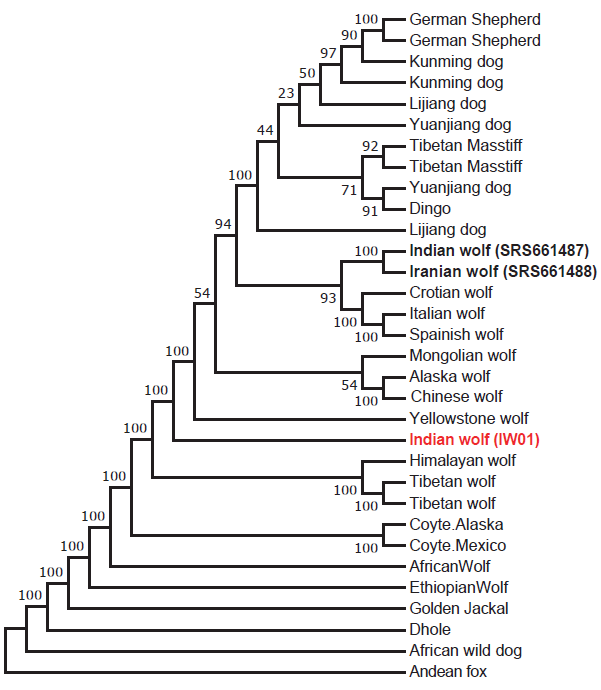


Figure S6: Neighbor-joining tree estimated from ‘diverged’ regions of ‘ghost’ origin (recovered by HMM; Wang, et al. 2020) in high-altitude (Himalayan and Tibetan) gray wolves. The domestic dog and gray wolf clades are poorly supported, which is likely due to the limited length of the regions (22.96Mb in total) used for neighbor-joining analysis.


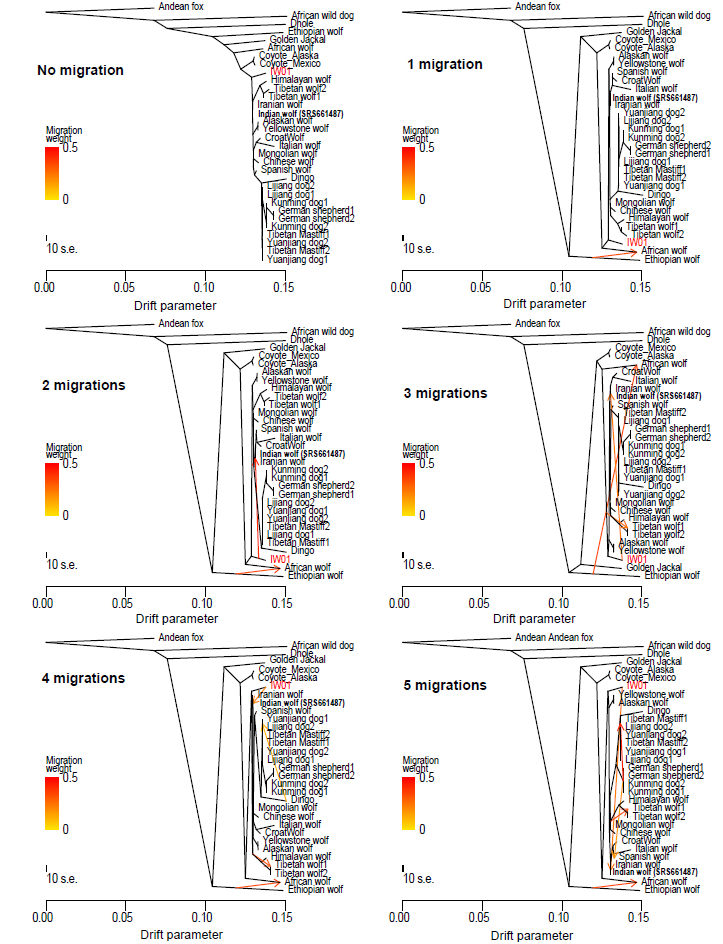


Figure S7: TreeMix tree graphs showing the migration and splitting of different canid taxa. In this plot, we present tree graphs inferred by allowing 0 to 5 migration edges.


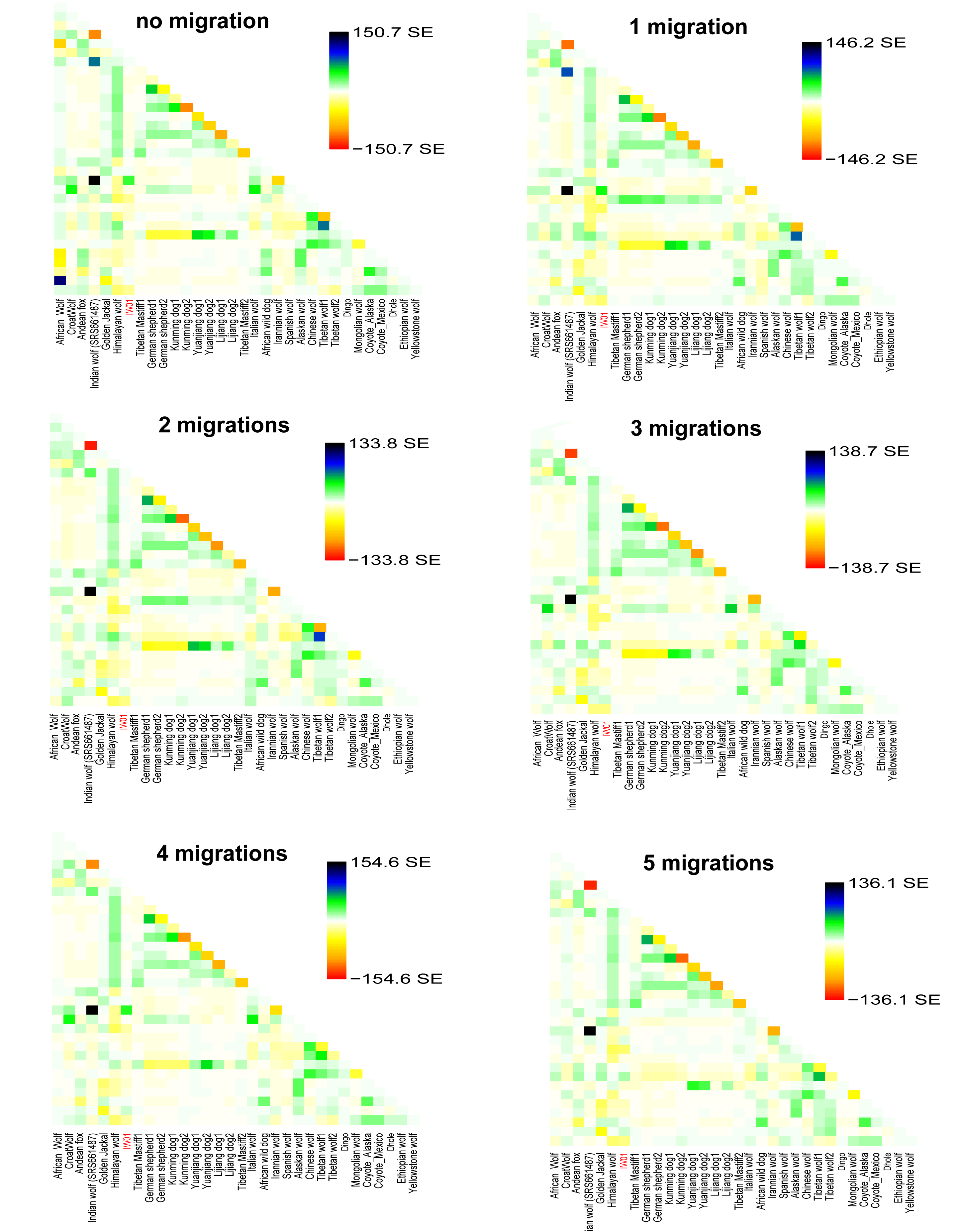


Figure S8: Treemix residual matrix for the graph by allowing 0 to 5 migrations shown in Figure S7


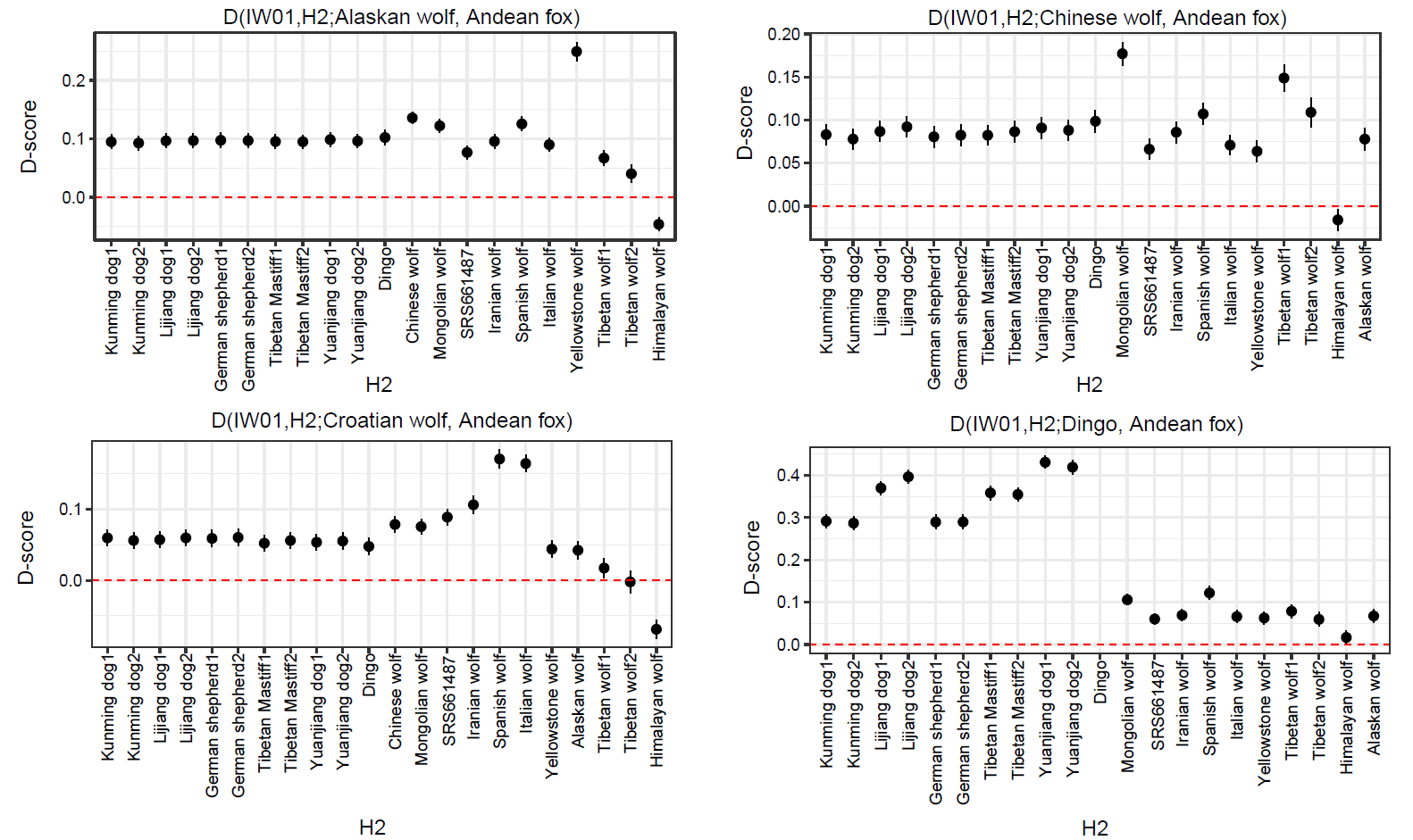


Figure S9: D-statistics analyses show that the domestic dog (represented here by the Dingo), Eurasian wolves, and North American wolves cluster as a clade sister to IW01 as D in the forms of D(IW01, H2; dog/Alaska/Chinese/European wolves, Andean fox) are significantly positive. D calculated when Himalayan or Tibetan wolves are in the H2 position showed negative values or smaller values, which may be due to high-altitude wolves carrying ancestries from a ghost lineage highly divergent from present lowland gray wolves and IW01 (Wang, et al. 2020 and this study).


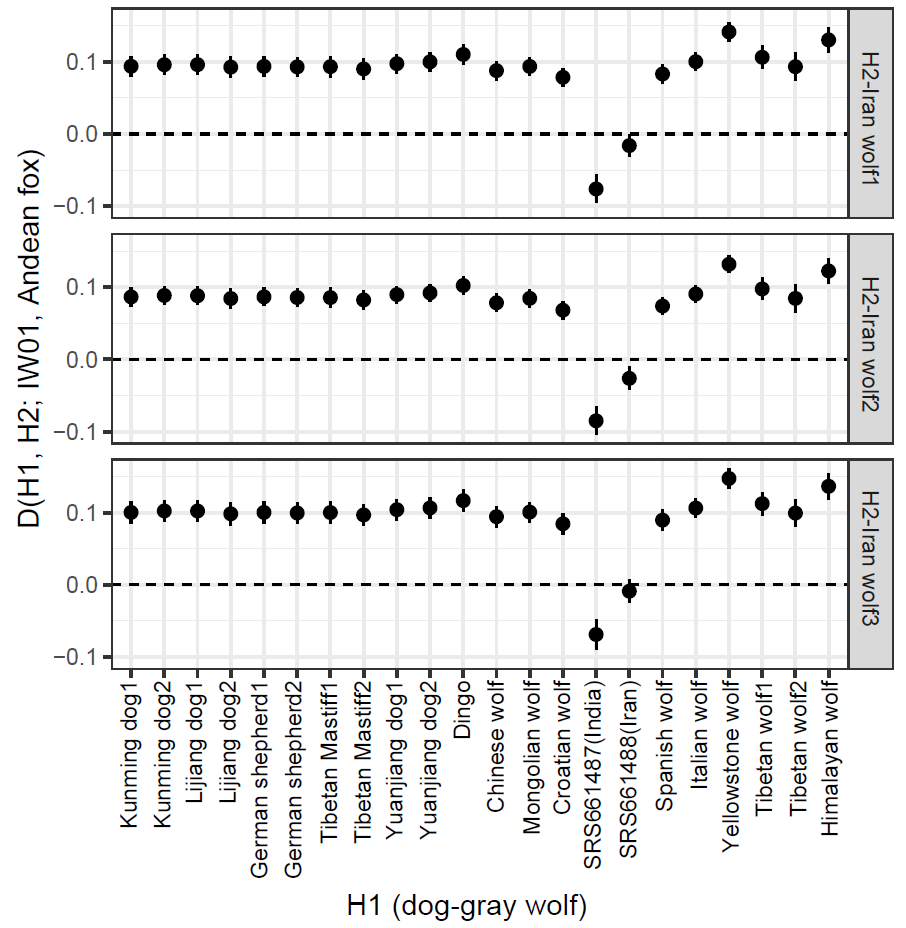


Figure S10: D-statistics in the forms of D(H1, H2; IW01, Andean fox) showing the amount of allele sharing between IW01 and each of three Iranian wolves (H2). The three Iranian wolf genomes (wolf1, 2 and 3) were retrieved from [https://ngdc.cncb.ac.cn/search/?dbId=gsa&q=CRA001324%20&page=1](https://ngdc.cncb.ac.cn/search/?dbId=gsa&q=CRA001324 &page=1).


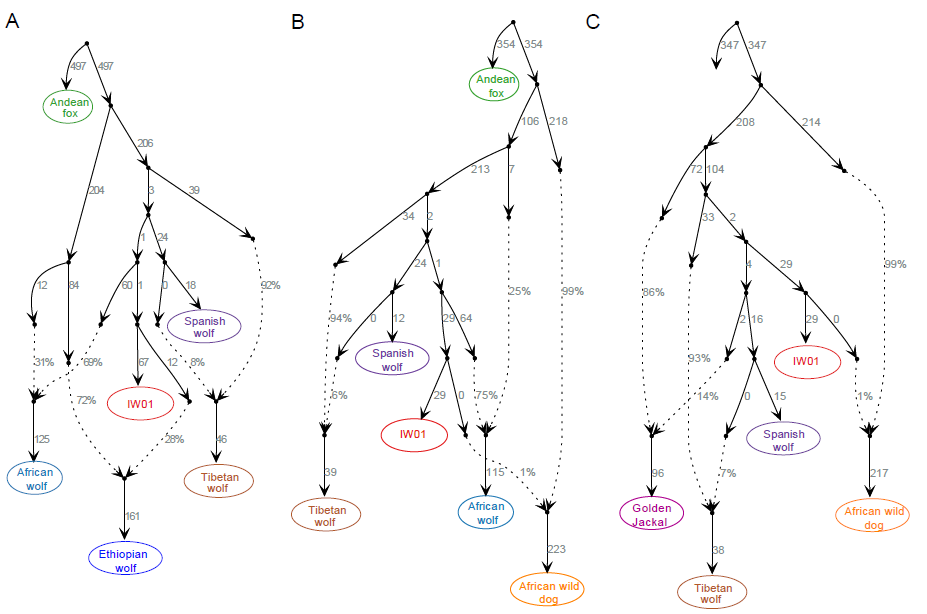


Figure S11: Admixture graphs (no f4 outliers) showing the genetic relationship among IW01, European wolves (represented here by the Spanish wolf), the high altitude Tibetan wolf, golden jackal, African wild dog. (A) This graph showing that both African wolf and Ethiopian wolf have admixed with IW01. (B) This graph showing that both African wolf and African wild dog have admixed with IW01. (C) This graph showing that African wild dog has admixed with IW01 and golden jackal carries admixed ancestries, 14% of which were from gray wolves. This result is consistent with the previous study ([Gopalakrishnan](https://www.ncbi.nlm.nih.gov/pubmed/?term=Gopalakrishnan S%5BAuthor%5D&cauthor=true&cauthor_uid=30344120) et al. 2018). IW01 has no gene flow with the golden jackal, consistent with our D-statistics analysis (**Figure 5G**). The golden jackal shows a signature of admixture with the ancestor of the domestic dog and gray wolves. Dashed lines indicate inferred admixture events and the admixture proportions are reported next to the dashed lines.


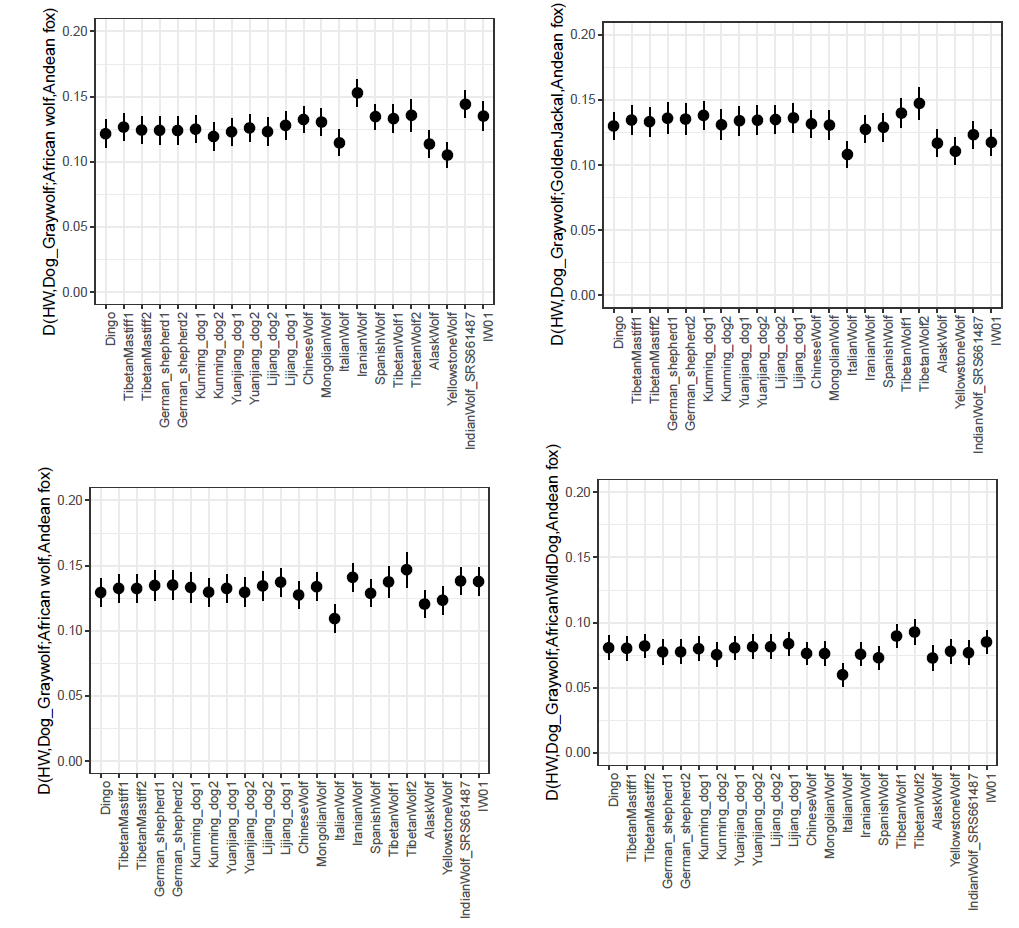


Figure. S12. D-statistics results showing African wild dog, Ethiopian wolf, African wolf, and golden jackal share an excess of alleles with domestic dog and gray wolves in comparison with the Himalayan wolf. All these D-statistics are statistically significant (Z > 3).


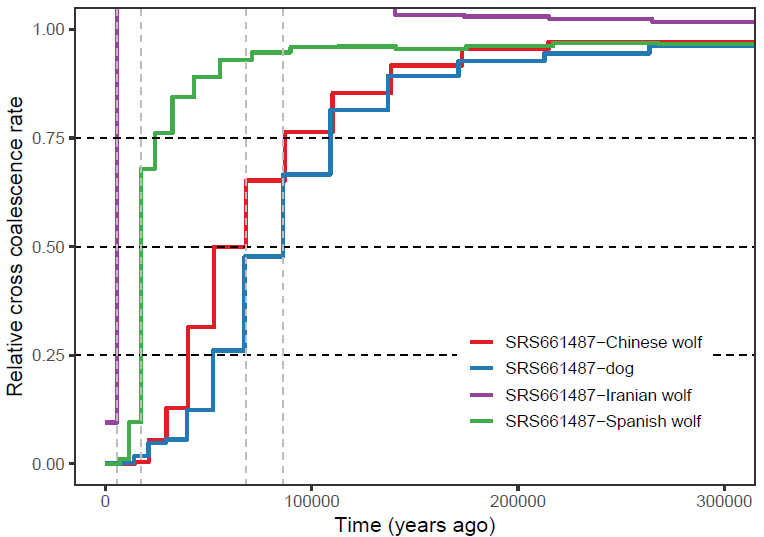


Figure S13: MSMC estimation (based on 50% of relative cross coalescence rate) of splitting time between SRS661487 (India) and the domestic dog and other gray wolves. Grey dashed lines indicate the estimated divergence time for each population pair.


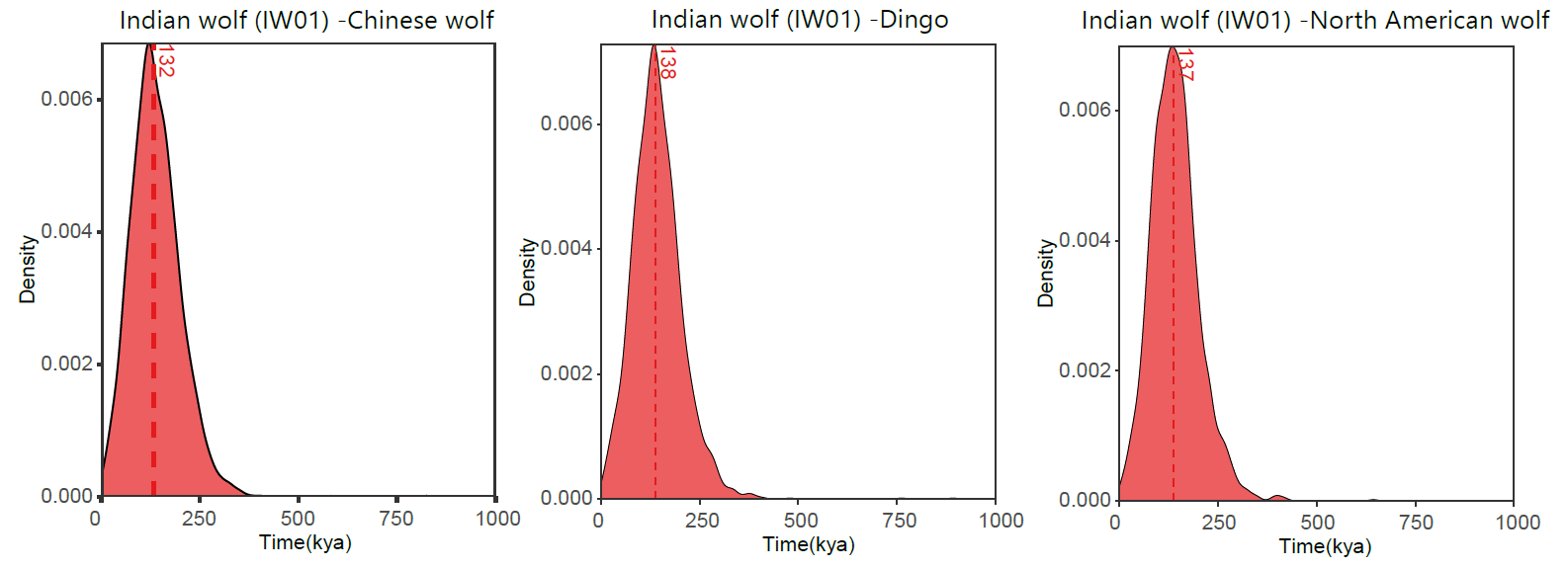


Figure S14: Coal-HMM estimation of divergence time between IW01 and dog and gray wolves.


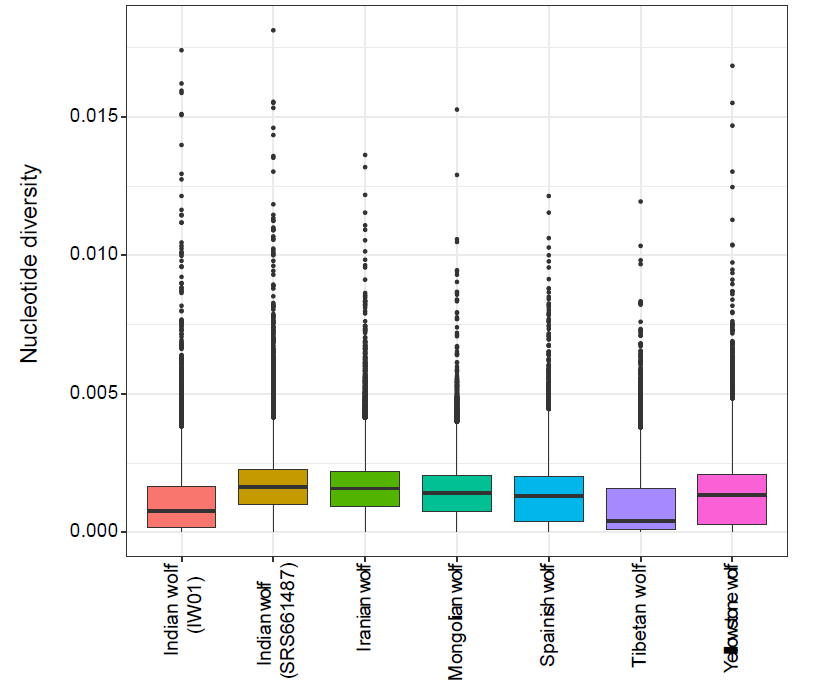


Figure S15: Nucleotide diversity among representatives of different gray wolf populations. This analysis was based on nuclear genomes with a minimum coverage depth >20-fold.


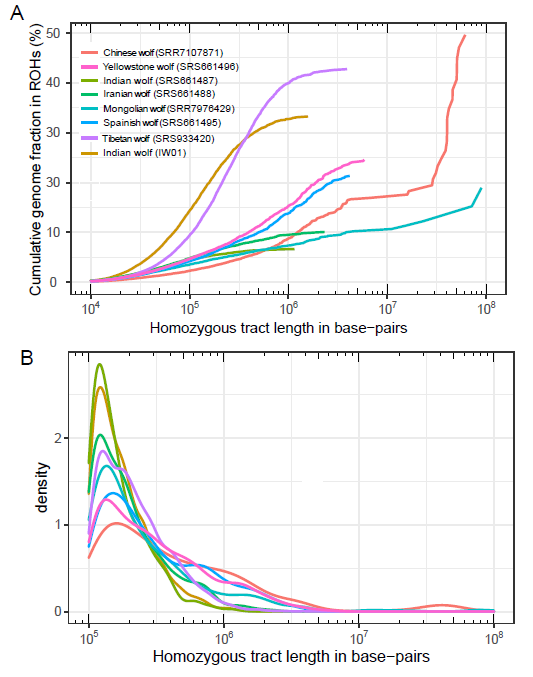


Figure S16. (A) : The cumulative proportion of the length of ROH across different gray wolf genomes. (B): The distribution of length of ROH (with length >=100kb being shown). Compared with other wolves, the Chinese wolf and Mongolian wolf have a greater number of larger ROH. SRS661487 (India) is likely the least inbred, with the least number of ROH >1Mb and the lowest proportion of ROH across the genome. The Chinese wolf and Mongolian wolf are highly inbred as they harbored 121 and 40 blocks of ROH with a length of >1Mb, respectively. Indeed, the Chinese wolf sample originated from a zoo animal and thus more likely to be inbred.

Table S1: Source and coverage information for the nuclear genomes used in this study

| **Species/breeds** | **NCBI-SRA accession IDs** | **Coverage (X)** | **Bio-Project accession IDs** | **References** |
| --- | --- | --- | --- | --- |
| Alaskan wolf | SRR8066602 | 9.28 | PRJNA496590 | [(Sinding, et al. 2018)](../../../../E:%5C00.WorkData%5C00.project%5CTibetan-dog_analysis%5CIndian-wolf%5C00.manuscript%5Cxx.xlsx#RANGE!_ENREF_54) |
| Yellowstone wolf | SRS661496 | 22.75 | [PRJNA255370](https://www.ncbi.nlm.nih.gov/bioproject/PRJNA255370) | [(Fan, et al. 2016)](../../../../E:%5C00.WorkData%5C00.project%5CTibetan-dog_analysis%5CIndian-wolf%5C00.manuscript%5Cxx.xlsx#RANGE!_ENREF_15) |
| Chinese wolf | SRR7107871 | 24.69 | [PRJNA274504](https://www.ncbi.nlm.nih.gov/bioproject/PRJNA274504) | [(Freedman, et al. 2014)](../../../../E:%5C00.WorkData%5C00.project%5CTibetan-dog_analysis%5CIndian-wolf%5C00.manuscript%5Cxx.xlsx#RANGE!_ENREF_17) |
| Mongolian wolf | SRR7976429 | 16.15 | PRJNA494719 | [(vonHoldt, et al. 2016)](../../../../E:%5C00.WorkData%5C00.project%5CTibetan-dog_analysis%5CIndian-wolf%5C00.manuscript%5Cxx.xlsx#RANGE!_ENREF_60) |
| Coyote.Alaska | SRR8049186 | 11.08 | [PRJNA494815](https://www.ncbi.nlm.nih.gov/bioproject/PRJNA494815) | [(Gopalakrishnan, et al. 2018)](../../../../E:%5C00.WorkData%5C00.project%5CTibetan-dog_analysis%5CIndian-wolf%5C00.manuscript%5Cxx.xlsx#RANGE!_ENREF_18) |
| Coyote.Mexico | SRR8049187 | 15.61 | [PRJNA494815](https://www.ncbi.nlm.nih.gov/bioproject/PRJNA494815) | [(Gopalakrishnan, et al. 2018)](../../../../E:%5C00.WorkData%5C00.project%5CTibetan-dog_analysis%5CIndian-wolf%5C00.manuscript%5Cxx.xlsx#RANGE!_ENREF_18) |
| Dhole | SRR8049189 | 17.66 | [PRJNA494815](https://www.ncbi.nlm.nih.gov/bioproject/PRJNA494815) | [(Gopalakrishnan, et al. 2018)](../../../../E:%5C00.WorkData%5C00.project%5CTibetan-dog_analysis%5CIndian-wolf%5C00.manuscript%5Cxx.xlsx#RANGE!_ENREF_18) |
| Dingo | SRR7120191 | 31.33 | [PRJNA448733](https://www.ncbi.nlm.nih.gov/bioproject/PRJNA448733) | [(Parker, et al. 2020)](../../../../E:%5C00.WorkData%5C00.project%5CTibetan-dog_analysis%5CIndian-wolf%5C00.manuscript%5Cxx.xlsx#RANGE!_ENREF_44) |
| German Shepherd1 | SRR1132487 | 12.28 | [PRJNA233638](https://www.ncbi.nlm.nih.gov/bioproject/PRJNA233638) | [(Gou, et al. 2014)](../../../../E:%5C00.WorkData%5C00.project%5CTibetan-dog_analysis%5CIndian-wolf%5C00.manuscript%5Cxx.xlsx#RANGE!_ENREF_19) |
| German Shepherd2 | SRR1132488 | 15.13 | [PRJNA233638](https://www.ncbi.nlm.nih.gov/bioproject/PRJNA233638) | [(Gou, et al. 2014)](../../../../E:%5C00.WorkData%5C00.project%5CTibetan-dog_analysis%5CIndian-wolf%5C00.manuscript%5Cxx.xlsx#RANGE!_ENREF_19) |
| Kunming dog1 | SRR1138310 | 13.26 | [PRJNA233638](https://www.ncbi.nlm.nih.gov/bioproject/PRJNA233638) | [(Gou, et al. 2014)](../../../../E:%5C00.WorkData%5C00.project%5CTibetan-dog_analysis%5CIndian-wolf%5C00.manuscript%5Cxx.xlsx#RANGE!_ENREF_19) |
| Kunming dog2 | SRR1138311 | 13.62 | [PRJNA233638](https://www.ncbi.nlm.nih.gov/bioproject/PRJNA233638) | [(Gou, et al. 2014)](../../../../E:%5C00.WorkData%5C00.project%5CTibetan-dog_analysis%5CIndian-wolf%5C00.manuscript%5Cxx.xlsx#RANGE!_ENREF_19) |
| Lijiang dog1 | SRR1138346 | 14.34 | [PRJNA233638](https://www.ncbi.nlm.nih.gov/bioproject/PRJNA233638) | [(Gou, et al. 2014)](../../../../E:%5C00.WorkData%5C00.project%5CTibetan-dog_analysis%5CIndian-wolf%5C00.manuscript%5Cxx.xlsx#RANGE!_ENREF_19) |
| Lijiang dog2 | SRR1138347 | 11.09 | [PRJNA233638](https://www.ncbi.nlm.nih.gov/bioproject/PRJNA233638) | [(Gou, et al. 2014)](../../../../E:%5C00.WorkData%5C00.project%5CTibetan-dog_analysis%5CIndian-wolf%5C00.manuscript%5Cxx.xlsx#RANGE!_ENREF_19) |
| Tibetan Mastiff1 | SRR1105792 | 14.1 | [PRJNA233638](https://www.ncbi.nlm.nih.gov/bioproject/PRJNA233638) | [(Gou, et al. 2014)](../../../../E:%5C00.WorkData%5C00.project%5CTibetan-dog_analysis%5CIndian-wolf%5C00.manuscript%5Cxx.xlsx#RANGE!_ENREF_19) |
| Tibetan Mastiff2 | SRR1138360 | 12.9 | [PRJNA233638](https://www.ncbi.nlm.nih.gov/bioproject/PRJNA233638) | [(Gou, et al. 2014)](../../../../E:%5C00.WorkData%5C00.project%5CTibetan-dog_analysis%5CIndian-wolf%5C00.manuscript%5Cxx.xlsx#RANGE!_ENREF_19) |
| Yuanjiang dog1 | SRR1138332 | 12.73 | [PRJNA233638](https://www.ncbi.nlm.nih.gov/bioproject/PRJNA233638) | [(Gou, et al. 2014)](../../../../E:%5C00.WorkData%5C00.project%5CTibetan-dog_analysis%5CIndian-wolf%5C00.manuscript%5Cxx.xlsx#RANGE!_ENREF_19) |
| Yuanjiang dog2 | SRR11383323 | 13.18 | [PRJNA233638](https://www.ncbi.nlm.nih.gov/bioproject/PRJNA233638) | [(Gou, et al. 2014)](../../../../E:%5C00.WorkData%5C00.project%5CTibetan-dog_analysis%5CIndian-wolf%5C00.manuscript%5Cxx.xlsx#RANGE!_ENREF_19) |
| Spanish wolf | SRS661495 | 22.04 | [PRJNA255370](https://www.ncbi.nlm.nih.gov/bioproject/PRJNA255370) | [(Fan, et al. 2016)](../../../../E:%5C00.WorkData%5C00.project%5CTibetan-dog_analysis%5CIndian-wolf%5C00.manuscript%5Cxx.xlsx#RANGE!_ENREF_15) |
| Croatian wolf | SRS1025420 | 9.66 | [PRJNA274504](https://www.ncbi.nlm.nih.gov/bioproject/PRJNA274504) | [(Freedman, et al. 2014)](../../../../E:%5C00.WorkData%5C00.project%5CTibetan-dog_analysis%5CIndian-wolf%5C00.manuscript%5Cxx.xlsx#RANGE!_ENREF_17) |
| Italian wolf | SRS661489 | 4.85 | [PRJNA255370](https://www.ncbi.nlm.nih.gov/bioproject/PRJNA255370) | [(Fan, et al. 2016)](../../../../E:%5C00.WorkData%5C00.project%5CTibetan-dog_analysis%5CIndian-wolf%5C00.manuscript%5Cxx.xlsx#RANGE!_ENREF_15) |
| Golden jackal | SRR2149878 | 9.08 | [PRJNA274504](https://www.ncbi.nlm.nih.gov/bioproject/PRJNA274504) | [(Freedman, et al. 2014)](../../../../E:%5C00.WorkData%5C00.project%5CTibetan-dog_analysis%5CIndian-wolf%5C00.manuscript%5Cxx.xlsx#RANGE!_ENREF_17) |
| Indian wolf | SRS661487 | 30.81 | [PRJNA255370](https://www.ncbi.nlm.nih.gov/bioproject/PRJNA255370) | [(Fan, et al. 2016)](../../../../E:%5C00.WorkData%5C00.project%5CTibetan-dog_analysis%5CIndian-wolf%5C00.manuscript%5Cxx.xlsx#RANGE!_ENREF_15) |
| Iranian wolf | SRS661488 | 24.39 | [PRJNA255370](https://www.ncbi.nlm.nih.gov/bioproject/PRJNA255370) | [(Fan, et al. 2016)](../../../../E:%5C00.WorkData%5C00.project%5CTibetan-dog_analysis%5CIndian-wolf%5C00.manuscript%5Cxx.xlsx#RANGE!_ENREF_15) |
| Tibetan wolf1 | SRS933415 | 22.6 | PRJNA448733 | [(Zhang, et al. 2014)](../../../../E:%5C00.WorkData%5C00.project%5CTibetan-dog_analysis%5CIndian-wolf%5C00.manuscript%5Cxx.xlsx#RANGE!_ENREF_68) |
| Tibetan wolf2 | SRS933420 | 20.58 | PRJNA448733 | [(Zhang, et al. 2014)](../../../../E:%5C00.WorkData%5C00.project%5CTibetan-dog_analysis%5CIndian-wolf%5C00.manuscript%5Cxx.xlsx#RANGE!_ENREF_68) |
| Himalayan wolf | SRS6146288 | 7.34 | [PRJNA559966](https://www.ncbi.nlm.nih.gov/bioproject/PRJNA559966) | [(W](../../../../E:%5C00.WorkData%5C00.project%5CTibetan-dog_analysis%5CIndian-wolf%5C00.manuscript%5Cxx.xlsx#RANGE!_ENREF_15)ang, et al. 2020) |
| Kenyan wild dog | SRS1188231 | 11.45 | [PRJNA304992](https://www.ncbi.nlm.nih.gov/bioproject/PRJNA304992) | [(Campana, et al. 2016)](../../../../E:%5C00.WorkData%5C00.project%5CTibetan-dog_analysis%5CIndian-wolf%5C00.manuscript%5Cxx.xlsx#RANGE!_ENREF_8) |
| African wolf (kenya) | ERS3334821 | 23.66 | [PRJEB31639](https://www.ncbi.nlm.nih.gov/bioproject/PRJEB31639) | [(Perri, et al. 2021)](../../../../E:%5C00.WorkData%5C00.project%5CTibetan-dog_analysis%5CIndian-wolf%5C00.manuscript%5Cxx.xlsx#RANGE!_ENREF_45) |
| Ethiopian wolf | SRR8049190 | 8.67 | [PRJNA494815](https://www.ncbi.nlm.nih.gov/bioproject/PRJNA494815) | [(Gopalakrishnan, et al. 2018)](../../../../E:%5C00.WorkData%5C00.project%5CTibetan-dog_analysis%5CIndian-wolf%5C00.manuscript%5Cxx.xlsx#RANGE!_ENREF_18) |
| Andean fox | [SRR1066702](https://trace.ncbi.nlm.nih.gov/Traces/sra/?run=SRR1066702) | 9.76 | [PRJNA232497](https://www.ncbi.nlm.nih.gov/bioproject/PRJNA232497) | [(Auton, et al. 2013)](../../../../E:%5C00.WorkData%5C00.project%5CTibetan-dog_analysis%5CIndian-wolf%5C00.manuscript%5Cxx.xlsx#RANGE!_ENREF_4) |
| Indian wolf(IW01) | SRR13985171 | 30.68 | PRJNA714797 | Generated in this study |

Tables S2: D-statistics in the form of D(H1, Northwestern African wolf; Ethiopian wolf, Andean fox) where H1 presents dog, gray wolf, and African wolf. When H1 presents dog and gray wolf, results showed an excess of allele sharing between Ethiopian wolf and Northwestern African wolf (as D< 0); when H1 presents Kenyan African wolf, it showed a signal of gene flow between Ethiopian wolf and Kenyan African wolf (as D > 0). Genomes for three northwestern African wolf (from Algerian, Morocco and Senegal) were from the previous study (Liu et al. 2018)

| H1 | H2 | H3 | ABBA | ABAB | D | SE | Z |
| --- | --- | --- | --- | --- | --- | --- | --- |
| African wolf (Algerian) | African wolf (Morocco) | EthopianWolf | 457566 | 471840 | -0.015 | 0.002 | -6.317 |
| African wolf (Algerian) | African wolf (Senegal) | EthopianWolf | 458022 | 441982 | 0.018 | 0.003 | 6.443 |
| African wolf (Morocco) | African wolf (Senegal) | EthopianWolf | 489709 | 458727 | 0.033 | 0.003 | 12.237 |
| African wolf (kenya) | African wolf (Morocco) | EthopianWolf | 488029 | 682037 | -0.166 | 0.004 | -43.334 |
| African wolf (kenya) | African wolf (Senegal) | EthopianWolf | 469704 | 626411 | -0.143 | 0.004 | -33.928 |
| African wolf (kenya) | African wolf (Algerian) | EthopianWolf | 465295 | 638581 | -0.157 | 0.004 | -40.654 |
| Alaskan wolf | African wolf (Morocco) | EthopianWolf | 623704 | 420026 | 0.195 | 0.003 | 65.412 |
| Alaskan wolf | African wolf (Senegal) | EthopianWolf | 618622 | 391184 | 0.225 | 0.003 | 74.464 |
| Alaskan wolf | African wolf (Algerian) | EthopianWolf | 601005 | 389952 | 0.213 | 0.003 | 73.393 |
| ChineseWolf | African wolf (Morocco) | EthopianWolf | 617614 | 416393 | 0.195 | 0.003 | 63.061 |
| ChineseWolf | African wolf (Senegal) | EthopianWolf | 611969 | 387252 | 0.225 | 0.003 | 73.487 |
| ChineseWolf | African wolf (Algerian) | EthopianWolf | 595359 | 387197 | 0.212 | 0.003 | 71.947 |
| Coyote.Alaska | African wolf (Morocco) | EthopianWolf | 680139 | 451777 | 0.202 | 0.003 | 67.844 |
| Coyote.Alaska | African wolf (Senegal) | EthopianWolf | 670805 | 418409 | 0.232 | 0.003 | 77.208 |
| Coyote.Alaska | African wolf (Algerian) | EthopianWolf | 655779 | 419683 | 0.220 | 0.003 | 76.365 |
| Coyote.Mexico | African wolf (Morocco) | EthopianWolf | 680779 | 453393 | 0.200 | 0.003 | 67.038 |
| Coyote.Mexico | African wolf (Senegal) | EthopianWolf | 671661 | 420029 | 0.230 | 0.003 | 74.436 |
| Coyote.Mexico | African wolf (Algerian) | EthopianWolf | 656049 | 421145 | 0.218 | 0.003 | 75.982 |
| Croatian wolf | African wolf (Morocco) | EthopianWolf | 588386 | 395865 | 0.196 | 0.003 | 64.607 |
| Croatian wolf | African wolf (Senegal) | EthopianWolf | 584144 | 368751 | 0.226 | 0.003 | 73.329 |
| Croatian wolf | African wolf (Algerian) | EthopianWolf | 567004 | 368022 | 0.213 | 0.003 | 72.782 |
| Dingo | African wolf (Morocco) | EthopianWolf | 626054 | 420682 | 0.196 | 0.003 | 61.065 |
| Dingo | African wolf (Senegal) | EthopianWolf | 619088 | 389966 | 0.227 | 0.003 | 71.714 |
| Dingo | African wolf (Algerian) | EthopianWolf | 602412 | 389918 | 0.214 | 0.003 | 70.361 |
| German Shepherd1 | African wolf (Morocco) | EthopianWolf | 622011 | 419191 | 0.195 | 0.003 | 60.640 |
| German Shepherd1 | African wolf (Senegal) | EthopianWolf | 615283 | 389080 | 0.225 | 0.003 | 71.664 |
| German Shepherd1 | African wolf (Algerian) | EthopianWolf | 598746 | 388957 | 0.212 | 0.003 | 68.629 |
| German Shepherd2 | African wolf (Morocco) | EthopianWolf | 623170 | 420030 | 0.195 | 0.003 | 60.901 |
| German Shepherd2 | African wolf (Senegal) | EthopianWolf | 615720 | 389270 | 0.225 | 0.003 | 73.703 |
| German Shepherd2 | African wolf (Algerian) | EthopianWolf | 599513 | 389355 | 0.213 | 0.003 | 68.820 |
| Himalayan wolf | African wolf (Morocco) | EthopianWolf | 680971 | 398743 | 0.261 | 0.003 | 89.899 |
| Himalayan wolf | African wolf (Senegal) | EthopianWolf | 674838 | 371562 | 0.290 | 0.003 | 98.527 |
| Himalayan wolf | African wolf (Algerian) | EthopianWolf | 659609 | 372200 | 0.279 | 0.003 | 93.928 |
| IranianWolf | African wolf (Morocco) | EthopianWolf | 608530 | 420566 | 0.183 | 0.003 | 58.978 |
| IranianWolf | African wolf (Senegal) | EthopianWolf | 602561 | 390476 | 0.214 | 0.003 | 69.251 |
| IranianWolf | African wolf (Algerian) | EthopianWolf | 586206 | 390277 | 0.201 | 0.003 | 68.410 |
| ItalianWolf | African wolf (Morocco) | EthopianWolf | 619014 | 408328 | 0.205 | 0.003 | 67.117 |
| ItalianWolf | African wolf (Senegal) | EthopianWolf | 614293 | 380621 | 0.235 | 0.003 | 77.751 |
| ItalianWolf | African wolf (Algerian) | EthopianWolf | 598002 | 380472 | 0.222 | 0.003 | 74.682 |
| IW01 | African wolf (Morocco) | EthopianWolf | 622397 | 427998 | 0.185 | 0.003 | 61.191 |
| IW01 | African wolf (Senegal) | EthopianWolf | 615615 | 397051 | 0.216 | 0.003 | 72.432 |
| IW01 | African wolf (Algerian) | EthopianWolf | 599275 | 396815 | 0.203 | 0.003 | 70.222 |
| Kunming dog1 | African wolf (Morocco) | EthopianWolf | 623907 | 418386 | 0.197 | 0.003 | 61.306 |
| Kunming dog1 | African wolf (Senegal) | EthopianWolf | 616744 | 387910 | 0.228 | 0.003 | 71.466 |
| Kunming dog1 | African wolf (Algerian) | EthopianWolf | 600778 | 388276 | 0.215 | 0.003 | 69.648 |
| Kunming dog2 | African wolf (Morocco) | EthopianWolf | 626698 | 420300 | 0.197 | 0.003 | 61.734 |
| Kunming dog2 | African wolf (Senegal) | EthopianWolf | 619328 | 389788 | 0.227 | 0.003 | 76.381 |
| Kunming dog2 | African wolf (Algerian) | EthopianWolf | 603492 | 390252 | 0.215 | 0.003 | 69.112 |
| Lijiang dog1 | African wolf (Morocco) | EthopianWolf | 615255 | 414173 | 0.195 | 0.003 | 62.114 |
| Lijiang dog1 | African wolf (Senegal) | EthopianWolf | 608229 | 384096 | 0.226 | 0.003 | 74.789 |
| Lijiang dog1 | African wolf (Algerian) | EthopianWolf | 591698 | 383803 | 0.213 | 0.003 | 68.650 |
| Lijiang dog2 | African wolf (Morocco) | EthopianWolf | 578719 | 389124 | 0.196 | 0.003 | 59.173 |
| Lijiang dog2 | African wolf (Senegal) | EthopianWolf | 571829 | 360872 | 0.226 | 0.003 | 69.833 |
| Lijiang dog2 | African wolf (Algerian) | EthopianWolf | 555130 | 360618 | 0.212 | 0.003 | 66.117 |
| MongolianWolf | African wolf (Morocco) | EthopianWolf | 611549 | 413234 | 0.194 | 0.003 | 63.698 |
| MongolianWolf | African wolf (Senegal) | EthopianWolf | 605765 | 383803 | 0.224 | 0.003 | 76.145 |
| MongolianWolf | African wolf (Algerian) | EthopianWolf | 589830 | 384125 | 0.211 | 0.003 | 71.256 |
| Indian wolf(SRS661487) | African wolf (Morocco) | EthopianWolf | 619100 | 424144 | 0.187 | 0.003 | 59.224 |
| Indian wolf(SRS661487) | African wolf (Senegal) | EthopianWolf | 612355 | 393081 | 0.218 | 0.003 | 71.594 |
| Indian wolf(SRS661487) | African wolf (Algerian) | EthopianWolf | 596364 | 393312 | 0.205 | 0.003 | 67.627 |
| SpanishWolf | African wolf (Morocco) | EthopianWolf | 612086 | 413705 | 0.193 | 0.003 | 63.347 |
| SpanishWolf | African wolf (Senegal) | EthopianWolf | 605288 | 383470 | 0.224 | 0.003 | 75.098 |
| SpanishWolf | African wolf (Algerian) | EthopianWolf | 588857 | 383523 | 0.211 | 0.003 | 71.987 |
| Tibetan Mastiff1 | African wolf (Morocco) | EthopianWolf | 624042 | 419979 | 0.195 | 0.003 | 60.814 |
| Tibetan Mastiff1 | African wolf (Senegal) | EthopianWolf | 617486 | 389745 | 0.226 | 0.003 | 71.681 |
| Tibetan Mastiff1 | African wolf (Algerian) | EthopianWolf | 601205 | 389941 | 0.213 | 0.003 | 68.892 |
| Tibetan Mastiff2 | African wolf (Morocco) | EthopianWolf | 621704 | 416619 | 0.198 | 0.003 | 63.201 |
| Tibetan Mastiff2 | African wolf (Senegal) | EthopianWolf | 616599 | 388083 | 0.227 | 0.003 | 76.015 |
| Tibetan Mastiff2 | African wolf (Algerian) | EthopianWolf | 599238 | 387072 | 0.215 | 0.003 | 71.492 |
| Tibetan wolf1 | African wolf (Morocco) | EthopianWolf | 621102 | 415043 | 0.199 | 0.003 | 63.827 |
| Tibetan wolf1 | African wolf (Senegal) | EthopianWolf | 614442 | 385560 | 0.229 | 0.003 | 73.968 |
| Tibetan wolf1 | African wolf (Algerian) | EthopianWolf | 598630 | 385963 | 0.216 | 0.003 | 71.259 |
| Tibetan wolf2 | African wolf (Morocco) | EthopianWolf | 620210 | 415117 | 0.198 | 0.003 | 61.825 |
| Tibetan wolf2 | African wolf (Senegal) | EthopianWolf | 612522 | 384671 | 0.228 | 0.003 | 72.248 |
| Tibetan wolf2 | African wolf (Algerian) | EthopianWolf | 597191 | 385387 | 0.216 | 0.003 | 69.889 |
| YellowstoneWolf | African wolf (Morocco) | EthopianWolf | 618516 | 416088 | 0.196 | 0.003 | 63.252 |
| YellowstoneWolf | African wolf (Senegal) | EthopianWolf | 613312 | 386584 | 0.227 | 0.003 | 75.259 |
| YellowstoneWolf | African wolf (Algerian) | EthopianWolf | 598402 | 388197 | 0.213 | 0.003 | 72.390 |
| Yuanjiang dog1 | African wolf (Morocco) | EthopianWolf | 623467 | 419647 | 0.195 | 0.003 | 60.783 |
| Yuanjiang dog1 | African wolf (Senegal) | EthopianWolf | 616711 | 389412 | 0.226 | 0.003 | 74.204 |
| Yuanjiang dog1 | African wolf (Algerian) | EthopianWolf | 600275 | 389457 | 0.213 | 0.003 | 68.986 |
| Yuanjiang dog2 | African wolf (Morocco) | EthopianWolf | 622968 | 415789 | 0.199 | 0.003 | 66.097 |
| Yuanjiang dog2 | African wolf (Senegal) | EthopianWolf | 617200 | 386511 | 0.230 | 0.003 | 77.033 |
| Yuanjiang dog2 | African wolf (Algerian) | EthopianWolf | 599777 | 385701 | 0.217 | 0.003 | 74.981 |

Table S3: D-statistics in the form of D(IW01, dog/wolf; Northwestern African wolf, Andean fox) showed gene flow between Northwestern African wolf and Indian gray wolf (IW01) as D < 0.

| H1 | H2 | H3 | ABBA | ABAB | D | SE | Z |
| --- | --- | --- | --- | --- | --- | --- | --- |
| IW01 | Alaskan wolf | African wolf (Algerian) | 544413 | 574893 | -0.027 | 0.003 | -9.120 |
| IW01 | Alaskan wolf | African wolf (Morocco) | 570678 | 600026 | -0.025 | 0.003 | -8.390 |
| IW01 | Alaskan wolf | African wolf (Senegal) | 539004 | 569724 | -0.028 | 0.003 | -9.432 |
| IW01 | Chinese wolf | African wolf (Algerian) | 536426 | 544207 | -0.007 | 0.003 | -2.450 |
| IW01 | Chinese wolf | African wolf (Morocco) | 562138 | 567682 | -0.005 | 0.003 | -1.741 |
| IW01 | Chinese wolf | African wolf (Senegal) | 531029 | 539060 | -0.008 | 0.003 | -2.767 |
| IW01 | Coyote.Alaska | African wolf (Algerian) | 539181 | 829921 | -0.212 | 0.005 | -42.677 |
| IW01 | Coyote.Alaska | African wolf (Morocco) | 561221 | 865190 | -0.213 | 0.005 | -44.188 |
| IW01 | Coyote.Alaska | African wolf (Senegal) | 539088 | 817361 | -0.205 | 0.005 | -43.014 |
| IW01 | Coyote.Mexico | African wolf (Algerian) | 540414 | 834841 | -0.214 | 0.005 | -43.176 |
| IW01 | Coyote.Mexico | African wolf (Morocco) | 563920 | 870927 | -0.214 | 0.005 | -43.583 |
| IW01 | Coyote.Mexico | African wolf (Senegal) | 539631 | 823080 | -0.208 | 0.005 | -43.158 |
| IW01 | Croatian wolf | African wolf (Algerian) | 513587 | 511367 | 0.002 | 0.003 | 0.758 |
| IW01 | Croatian wolf | African wolf (Morocco) | 536968 | 534071 | 0.003 | 0.003 | 0.967 |
| IW01 | Croatian wolf | African wolf (Senegal) | 507750 | 508229 | 0.000 | 0.003 | -0.171 |
| IW01 | Dingo | African wolf (Algerian) | 539640 | 560335 | -0.019 | 0.003 | -6.083 |
| IW01 | Dingo | African wolf (Morocco) | 564740 | 586047 | -0.019 | 0.003 | -6.260 |
| IW01 | Dingo | African wolf (Senegal) | 534292 | 554585 | -0.019 | 0.003 | -6.363 |
| IW01 | German Shepherd1 | African wolf (Algerian) | 536235 | 550033 | -0.013 | 0.003 | -4.209 |
| IW01 | German Shepherd1 | African wolf (Morocco) | 559885 | 575426 | -0.014 | 0.003 | -4.659 |
| IW01 | German Shepherd1 | African wolf (Senegal) | 529903 | 544638 | -0.014 | 0.003 | -4.609 |
| IW01 | German Shepherd2 | African wolf (Algerian) | 538775 | 550176 | -0.010 | 0.003 | -3.379 |
| IW01 | German Shepherd2 | African wolf (Morocco) | 561578 | 576285 | -0.013 | 0.003 | -4.357 |
| IW01 | German Shepherd2 | African wolf (Senegal) | 532228 | 545256 | -0.012 | 0.003 | -3.982 |
| IW01 | Himalayan wolf | African wolf (Algerian) | 476487 | 618516 | -0.130 | 0.004 | -32.855 |
| IW01 | Himalayan wolf | African wolf (Morocco) | 504811 | 642126 | -0.120 | 0.004 | -33.927 |
| IW01 | Himalayan wolf | African wolf (Senegal) | 472173 | 613877 | -0.130 | 0.004 | -35.546 |
| IW01 | Iranian wolf | African wolf (Algerian) | 522078 | 503089 | 0.019 | 0.003 | 5.564 |
| IW01 | Iranian wolf | African wolf (Morocco) | 545063 | 525687 | 0.018 | 0.003 | 6.394 |
| IW01 | Iranian wolf | African wolf (Senegal) | 517845 | 498473 | 0.019 | 0.003 | 5.704 |
| IW01 | Italian wolf | African wolf (Algerian) | 527085 | 544530 | -0.016 | 0.003 | -5.362 |
| IW01 | Italian wolf | African wolf (Morocco) | 558114 | 568103 | -0.009 | 0.003 | -3.049 |
| IW01 | Italian wolf | African wolf (Senegal) | 520743 | 540203 | -0.018 | 0.003 | -6.043 |
| IW01 | Kunming dog1 | African wolf (Algerian) | 535972 | 550785 | -0.014 | 0.003 | -4.450 |
| IW01 | Kunming dog1 | African wolf (Morocco) | 560877 | 574675 | -0.012 | 0.003 | -4.108 |
| IW01 | Kunming dog1 | African wolf (Senegal) | 530894 | 544956 | -0.013 | 0.003 | -4.486 |
| IW01 | Kunming dog2 | African wolf (Algerian) | 537768 | 553262 | -0.014 | 0.003 | -4.630 |
| IW01 | Kunming dog2 | African wolf (Morocco) | 562531 | 578629 | -0.014 | 0.003 | -4.761 |
| IW01 | Kunming dog2 | African wolf (Senegal) | 531988 | 548706 | -0.015 | 0.003 | -5.220 |
| IW01 | Lijiang dog1 | African wolf (Algerian) | 531401 | 543701 | -0.011 | 0.003 | -3.739 |
| IW01 | Lijiang dog1 | African wolf (Morocco) | 555827 | 568710 | -0.011 | 0.003 | -3.883 |
| IW01 | Lijiang dog1 | African wolf (Senegal) | 525337 | 538603 | -0.012 | 0.003 | -4.247 |
| IW01 | Lijiang dog2 | African wolf (Algerian) | 498033 | 508445 | -0.010 | 0.003 | -3.197 |
| IW01 | Lijiang dog2 | African wolf (Morocco) | 522218 | 534460 | -0.012 | 0.003 | -3.795 |
| IW01 | Lijiang dog2 | African wolf (Senegal) | 492353 | 504425 | -0.012 | 0.003 | -4.125 |
| IW01 | MongolianWolf | African wolf (Algerian) | 531016 | 539662 | -0.008 | 0.003 | -2.630 |
| IW01 | MongolianWolf | African wolf (Morocco) | 558052 | 563029 | -0.004 | 0.003 | -1.451 |
| IW01 | MongolianWolf | African wolf (Senegal) | 525766 | 533962 | -0.008 | 0.003 | -2.530 |
| IW01 | Indian wolf(SRS661487) | African wolf (Algerian) | 495815 | 489010 | 0.007 | 0.003 | 2.298 |
| IW01 | Indian wolf(SRS661487) | African wolf (Morocco) | 518682 | 511386 | 0.007 | 0.003 | 2.618 |
| IW01 | Indian wolf(SRS661487) | African wolf (Senegal) | 491372 | 484528 | 0.007 | 0.003 | 2.429 |
| IW01 | SpanishWolf | African wolf (Algerian) | 534625 | 533952 | 0.001 | 0.003 | 0.227 |
| IW01 | SpanishWolf | African wolf (Morocco) | 559071 | 559169 | 0.000 | 0.003 | -0.032 |
| IW01 | SpanishWolf | African wolf (Senegal) | 528752 | 528794 | 0.000 | 0.003 | -0.014 |
| IW01 | Tibetan Mastiff1 | African wolf (Algerian) | 536610 | 552944 | -0.015 | 0.003 | -4.718 |
| IW01 | Tibetan Mastiff1 | African wolf (Morocco) | 562753 | 576729 | -0.012 | 0.003 | -4.060 |
| IW01 | Tibetan Mastiff1 | African wolf (Senegal) | 531538 | 546693 | -0.014 | 0.003 | -5.024 |
| IW01 | Tibetan Mastiff2 | African wolf (Algerian) | 535252 | 548522 | -0.012 | 0.003 | -4.031 |
| IW01 | Tibetan Mastiff2 | African wolf (Morocco) | 559628 | 572638 | -0.011 | 0.003 | -4.083 |
| IW01 | Tibetan Mastiff2 | African wolf (Senegal) | 527172 | 544359 | -0.016 | 0.003 | -5.742 |
| IW01 | Tibetan wolf1 | African wolf (Algerian) | 523549 | 557832 | -0.032 | 0.003 | -9.782 |
| IW01 | Tibetan wolf1 | African wolf (Morocco) | 550658 | 582793 | -0.028 | 0.003 | -8.862 |
| IW01 | Tibetan wolf1 | African wolf (Senegal) | 520436 | 552762 | -0.030 | 0.003 | -9.783 |
| IW01 | Tibetan wolf2 | African wolf (Algerian) | 511792 | 554128 | -0.040 | 0.004 | -10.860 |
| IW01 | Tibetan wolf2 | African wolf (Morocco) | 537258 | 578927 | -0.037 | 0.003 | -10.862 |
| IW01 | Tibetan wolf2 | African wolf (Senegal) | 509178 | 548026 | -0.037 | 0.003 | -10.519 |
| IW01 | YellowstoneWolf | African wolf (Algerian) | 535371 | 569294 | -0.031 | 0.003 | -10.054 |
| IW01 | YellowstoneWolf | African wolf (Morocco) | 559232 | 592579 | -0.029 | 0.003 | -9.942 |
| IW01 | YellowstoneWolf | African wolf (Senegal) | 531922 | 562816 | -0.028 | 0.003 | -9.833 |
| IW01 | Yuanjiang dog1 | African wolf (Algerian) | 538174 | 553203 | -0.014 | 0.003 | -4.482 |
| IW01 | Yuanjiang dog1 | African wolf (Morocco) | 561265 | 577898 | -0.015 | 0.003 | -5.039 |
| IW01 | Yuanjiang dog1 | African wolf (Senegal) | 530444 | 547773 | -0.016 | 0.003 | -5.693 |
| IW01 | Yuanjiang dog2 | African wolf (Algerian) | 539801 | 551461 | -0.011 | 0.003 | -3.420 |
| IW01 | Yuanjiang dog2 | African wolf (Morocco) | 565209 | 576515 | -0.010 | 0.003 | -3.276 |
| IW01 | Yuanjiang dog2 | African wolf (Senegal) | 532575 | 546955 | -0.013 | 0.003 | -4.651 |
| African wolf (Morocco) | African wolf (Senegal) | IW01 | 659093 | 678416 | -0.014 | 0.003 | -4.996 |
| African wolf (Kenya) | African wolf (Algerian) | IW01 | 828812 | 596989 | 0.163 | 0.003 | 53.144 |
| African wolf (Kenya) | African wolf (Morocco) | IW01 | 871231 | 637736 | 0.155 | 0.003 | 50.946 |
| African wolf (Kenya) | African wolf (Senegal) | IW01 | 804267 | 598165 | 0.147 | 0.003 | 49.185 |
| African wolf (Algerian) | African wolf (Morocco) | IW01 | 656420 | 662502 | -0.005 | 0.003 | -1.485 |
| African wolf (Algerian) | African wolf (Senegal) | IW01 | 624533 | 649297 | -0.019 | 0.003 | -6.452 |

Table S4: Total number and length of potentially admixed tracts between IW01 and each of three African canid species.

| **Window size** | **Terms** | **African wolf** | **Ethiopian wolf** | **African wild dog** |
| --- | --- | --- | --- | --- |
| -w 40 | # hap blocks | 3085 | 1174 | 78 |
| -w 40 | Blocks length (mean: bp) | 13998 | 8476 | 5515 |
| -w 20 | # hap blocks | 5611 | 1856 | 186 |
| -w 20 | Blocks length (mean: bp) | 6540 | 3914 | 2286 |
